# Supplementary material for: Changes in lipoproteins associated with lipid-lowering and antiplatelet strategies in patients with acute myocardial infarction
Source: PLoS One. 2022 Aug 30;17(8):e0273292. doi: 10.1371/journal.pone.0273292 (PMC9426937; doi:10.1371/journal.pone.0273292)
Supplement: S1 File — (DOCX) [file pone.0273292.s003.docx]

**PROJETO TEMÁTICO**

**PAPEL DA IMUNIDADE INATA E ADAPTATIVA NA CARDIOPATIA ISQUEMICA APÓS INFARTO AGUDO DO MIOCÁRDIO**

**Pesquisador responsável:**

Francisco Antonio Helfenstein Fonseca

Setor de Lípides, Aterosclerose e Biologia Vascular

Disciplina de Cardiologia

Escola Paulista de Medicina

Universidade Federal de São Paulo

2012

**1. RESUMO**

Durante centenas de milhares de anos, parte de nosso patrimônio genômico foi primariamente desenvolvido para combate a agentes infecciosos. Entretanto, a participação de nosso sistema imune modulando a evolução da aterosclerose foi um desafio muito mais recentemente reconhecido. O fascinante e diferenciado papel de subtipos de linfócitos na evolução da doença coronária poderá se constituir em novo alvo estratégico para compreensão e terapia do infarto do miocárdio. A perda muscular nas primeiras horas da oclusão coronária determina importante papel prognóstico e a reperfusão coronária com trombolíticos ou intervenção percutânea constituem estratégias de eleição para estes pacientes. Entretanto, ao redor da área necrótica, forma-se uma área isquêmica que evolui para extensão do infarto ou se recupera, principalmente nas três semanas seguintes ao insulto isquêmico. São desconhecidos os determinantes da viabilidade celular, postulando-se que decorram de fatores não apenas relacionados à microcirculação ou gasto energético, mas dependente da resposta inflamatória e imune. Além disso, ciclos de mobilização de linfócitos secundários ao infarto determinam colonização de placas ateroscleróticas em diferentes estágios de desenvolvimento, contribuindo para recorrência precoce de desfechos isquêmicos. Este projeto visa maior entendimento dos mecanismos imunes e metabólicos envolvidos na recuperação do miocárdio isquêmico e progressão da doença coronária. Especificamente, estudaremos a imunidade inata e adaptativa, com ênfase em subtipos de linfócitos envolvidos na evolução precoce e tardia de pacientes com infarto agudo do miocárdio e relacionados com a oxidação da LDL. O projeto ainda envolve a quantificação de biomarcadores identificados por estudos de metabolômica, bem como vias de sinalização celular envolvidas na evolução da doença. Terapêuticas farmacológicas e mudanças na flora intestinal serão examinadas desde a fase aguda do infarto do miocárdio até seis meses de evolução clínica, com base em resultados de estudos de ressonância nuclear magnética com realce tardio na fase aguda e tardia do infarto do miocárdio.

**ROLE OF INNATE AND ADAPTIVE IMMUNITY ON THE ISCHEMIC HEART DISEASE AFTER ACUTE MYOCARDIAL INFARCTION**

**1. ABSTRACT**

For hundreds of thousands of years, part of our genomic heritage was primarily developed to combat infectious agents. However, our immune system modulating the progression of atherosclerosis was a challenge much more recently recognized. The fascinating and differential role of lymphocyte subtypes in the development of coronary artery disease may be a new strategic target for understanding and therapy of acute myocardial infarction. The muscle loss in the first hours of coronary occlusion determines important prognostic role and the coronary reperfusion either with thrombolytic agents or percutaneous intervention are currently the strategies of choice for these patients. However, surrounding the necrotic core, an ischemic area is formed, that progresses to extension of the infarction or recovers, mainly during the next three weeks after the ischemic insult. The determinants of cell viability are unknown, postulating that they arise from factors not only related to microcirculation or energy expenditure, but also to inflammatory and immune responses. Furthermore, the intense mobilization of progenitor cells secondary to myocardial infarction triggers large lymphocyte mobilization that colonizes plaques in development, contributing to recurrence of ischemic events. This project aims to understanding the immune and metabolic mechanisms involved in the recovery of the ischemic myocardium and coronary disease progression. Specifically, we will study the innate and adaptive immunity, with emphasis on lymphocytes subtypes involved in the coronary heart disease of patients with acute myocardial infarction. In addition, the project will evaluate biomarkers identified by metabolomics studies, as well as the corresponding signaling pathways. Therapeutic pharmacological strategies and changes on intestine microflora will be evaluated since the acute phase of myocardial infarction up to six months, based on the MRI studies with late enhancement performed in the acute and late follow-up.

**2. ENUNCIADO DO PROBLEMA**

Este projeto permitirá o aprofundamento das pesquisas do Laboratório de Lípides, Aterosclerose e Biologia Vascular da Escola Paulista de Medicina, Universidade Federal de São Paulo, na compreensão de mecanismos envolvidos na reparação tecidual e recuperação do miocárdio isquêmico após o infarto agudo do miocárdio.

O Setor de Lípides, Aterosclerose e Biologia Vascular é um setor da Disciplina de Cardiologia da EPM/Unifesp criado em 1998. O grupo desenvolve estudos experimentais e ensaios clínicos desde sua criação, integrando médicos, biólogos, biomédicos, educadores físicos, nutricionistas, químicos, físicos, biofísicos, veterinários e fisioterapeutas, permitindo uma abordagem multidisciplinar em suas pesquisas. Com estas características, o grupo estabeleceu sólidas interfaces, permitindo o uso de ferramentas progressivamente mais sofisticadas na compreensão da aterosclerose, sua patogenia e mecanismos terapêuticos envolvidos no controle de sua de evolução.

Pesquisas no entendimento da reparação endotelial após lesão por cateter-balão tiveram início com trabalhos experimentais desenvolvidos no Departamento de Biofísica da EPM/Unifesp em ratos e a seguir nos EUA (The Mount Sinai School of Medicine) em suínos com prosseguimento no Setor de Lípides, Aterosclerose e Biologia Vascular na Unifesp, pela utilização do modelo de aterosclerose em coelhos para estudos de lesão vascular e vasorreatividade endotelial em anéis isolados (banho de órgãos) e *in vivo* (angiografia coronária)^1-4^.

Estudos clínicos de vasorreatividade constituíram o segundo estágio de envolvimento nas pesquisas sobre função endotelial^5,6^ e a dilatação mediada pelo fluxo foi melhor compreendida quando passamos a examinar o *turnover* endotelial com quantificação de células progenitoras endoteliais e particularmente, micropartículas endoteliais e plaquetárias^7,8^. Paralelamente, estudos morfológicos experimentais da aterosclerose foram desenvolvidos, envolvendo estratégias terapêuticas^9-12^.

Nos últimos anos, incorporamos a visão da aterosclerose como doença inflamatória e imune^13-23^. Neste contexto, por meio de participação em dois institutos (Instituto Milênio de Fluidos Complexos e o Instituto Nacional de Ciência e Tecnologia de Fluidos Complexos - INCT-FCx) passamos a estudar mais detalhadamente a resposta imune deflagrada pela oxidação de lipoproteínas e o balanço entre síntese e absorção de colesterol, na dependência da estratégia terapêutica instituída (farmacológica e não farmacológica)^24-28^.

Ensaios clínicos, estudos do tipo caso e controle e aqueles envolvendo estratégias terapêuticas ou estudos observacionais em longo prazo foram desenvolvidos, e em muitos destes tivemos a oportunidade de estudar variantes genéticas e utilizar novas ferramentas da biologia molecular, como sequenciamento gênico e RT-PCR em tempo real^29-58^.

Mais recentemente, passamos a estudar interações farmacocinéticas entre medicamentos comumente empregados na fase aguda de síndromes coronarianas, e encontramos importantes interações entre tienopiridínicos e estatinas^8,59^, explorando seus efeitos pleiotrópicos nas primeiras horas de administração dos fármacos, como a notável melhora da função endotelial, aumento das concentrações séricas das estatinas e maior concentração de clopidogrel relacionadas de maneira inversa com as concentrações plasmáticas de micropartículas plaquetárias. Trabalhos mais recentes de nosso grupo sugeriram grande potencial de atuação destes fármacos na microcirculação, protegendo o miocárdio isquêmico, diminuindo o consumo e apoptose de plaquetas e células endoteliais, bem como ampliando a mobilização de células progenitoras da medula, favorecendo a reparação tecidual.

A aterosclerose é hoje compreendida como doença inflamatória e autoimune crônica, envolvendo respostas dos compartimentos celulares e humorais da imunidade inata e adaptativa. Estas respostas estão demonstradas pelos autoanticorpos e complexos imunes presentes nas lesões ateroscleróticas^60^. As respostas da imunidade inata são principalmente decorrentes da participação de monócitos/macrófagos e células dendríticas, em placas iniciais e avançadas^61^. Estas células são geralmente pró-aterogênicas, embora alguns subtipos tenham papel antiaterogênico^62^. De forma interessante, células B1 encontradas em vários tecidos como baço, intestino, peritôneo e cavidades pleurais podem se diferenciar em células B1a e B1b, além de Breg e B10^63^. As células dendríticas interagem na diferenciação de células T em subtipos específicos (Th1, Th2, Treg) que determinam respostas pró-aterogênicas (Th1) ou protetoras como o Treg^64^.

Um aspecto notável foi a demonstração de que a adventícia contém grande população de células B, formando o chamado tecido linfóide terciário^65^. Enquanto as células B1 se mostraram ateroprotetoras, principalmente por produzirem IgM anti-LDLoxidadas e facilitarem o seu *clearance*, evitando a exposição das destas às células dendríticas e subsequente ativação da imunidade celular, as células B2 foram consideradas pró-aterogênicas, por induzirem a produção de IgG associadas à deposição de imunocomplexos na íntima vascular^66^.

Mais recentemente, o efluxo de macrófagos da íntima vascular foi descrito, envolvendo sinalização relacionada às quimiocinas CCL19 e CCL21 e o receptor CCR7, possivelmente via *vasa vasorum*^67,68^. Fica evidente que a presença de células B neste sítio vascular esteja implicada na defesa fisiológica do organismo, associada à neovascularização do vaso e que o efluxo de macrófagos na íntima vascular constitua processo dinâmico, mediado na adventícia por células imunes.

Foi ainda sugerido que a elevação do colesterol diminua o efluxo de macrófagos, favorecendo sua residência na íntima vascular e subseqüentes complicações da placa, adicionando novos benefícios potenciais para as estatinas^69^. Finalmente, a injeção de células B em modelo experimental de infarto do miocárdio mostrou-se efetiva para reparação do tecido isquêmico e melhor remodelamento ventricular^70^.

Existe grande recorrência de desfechos cardiovasculares após o infarto do miocárdio e, neste sentido, a mobilização de células progenitoras medulares, em resposta à lesão miocárdica determina maior colonização de linfócitos em placas já estabelecidas em outros sítios vasculares, o que explica a maior vulnerabilidade destas para novas complicações trombóticas, estabelecendo importante elo fisiopatológico a explicar a alta recorrência de eventos ao longo do primeiro ano após uma síndrome coronariana aguda^71^.

Nosso propósito neste projeto temático é o de caracterizar subtipos de linfócitos B e determinar seu papel no remodelamento ventricular em curto e longo prazo na evolução da doença coronária após infarto agudo do miocárdio. Além disso, iremos explorar estratégias de proteção da microcirculação e seus efeitos precoces e tardios na massa infartada (ressonância nuclear magnética com realce tardio) e função ventricular. Recentemente, mostramos diferenças na quantificação de IgG total para LDLoxidada na comparação da rosuvastatina com a sinvastatina + ezetimiba, a despeito do mesmo grau de redução do LDL-colesterol^72^. Em projeto piloto, recentemente quantificamos linfócitos B1(CD70-CD19+CD20+CD27+) e B2 (CD23+CD19+), além de linfócitos T CD4 e CD8, com amostras colhidas nas primeiras horas do infarto agudo do miocárdio, e comparamos com controles saudáveis, encontrando aumento significante de B2 e tendências para aumento de TCD4 e reduções de B1 e TCD8, mostrando dados promissores relacionando a maior presença de linfócitos B2, que poderá deflagrar mecanismos para aterosclerose e vulnerabilidade de placas se mantido em longo prazo^73,74^ .

Dentre estas estratégias, examinaremos o sinergismo entre estatinas e antiplaquetários, ao lado de mudanças da microbiota e seus efeitos na diferenciação de linfócitos, evolução da cardiopatia isquêmica, parâmetros bioquímicos e nos analitos de metabolômica. Os subestudos descritos a seguir mostram a fundamentação teórica destas intervenções, sua originalidade e relevância clínica.

***SUBPROJETO 1***

**Efeitos de interações entre estatinaS e antiplaquetários NA EVOLUÇÃO DO INFARTO AGUDO DO MIOCÁRDIO**

**Resumo**

A oclusão coronária determina perda de massa muscular miocárdica nas primeiras horas do infarto agudo do miocárdio (IAM), mas ao redor desta, uma zona de miocárdio isquêmico pode se recuperar ou evoluir para aumento da massa infartada, ao longo das primeiras três ou quatro semanas, com implicações prognósticas em longo prazo. Recuperação funcional do endotélio parece fundamental para melhora da microcirculação e benefícios no remodelamento ventricular. Este subprojeto irá examinar possíveis benefícios da interação farmacocinética sinérgica entre estatinas e antiplaquetários nas ações antitrombóticas e vasodilatadoras. Pacientes com IAM com supradesnível de segmento ST, tratados nas primeiras seis horas do infarto com trombolíticos ou intervenção percutânea, serão aleatorizados em desenho fatorial 2x2 para tratamento com rosuvastatina 20 mg/dia ou sinvastatina 40 mg e ezetimiba 10 mg diariamente, bem como ticagrelor 180 mg inicialmente e 90 mg 2x/dia de manutenção ou clopidogrel 600 mg inicialmente e 75 mg/dia de manutenção (pacientes com estratégia de intervenção percutânea primária) ou 90 mg de ticagrelor (dose inicial e mantida 12/12h) e 300 mg de clopidogrel (seguido de 75 mg/dia, quando do uso de trombolíticos), além da terapia convencional do IAM. O tratamento será mantido por 6 meses e durante este período, exames de ressonância nuclear magnética com realce tardio (RNM-RT) serão realizados entre o 3° e 5° dias pós IAM e repetidos após quatro semanas e seis meses, para quantificação da necrose miocárdica, tecido isquêmico e exame da função ventricular esquerda.

**1. Fundamentação da proposta**

O primeiro estudo com estatina, envolvendo pacientes coronarianos agudos para análise de desfechos clínicos, incluíu pacientes com IAM sem supradesnível de segmento ST, mas a atorvastatina foi administrada apenas no período entre 24 e 96 horas de IAM^75^. A seguir, o estudo PROVE-IT^76^, comparando atorvastatina e pravastatina, examinou a intervenção de forma ainda mais tardia, a partir do 10° dia do IAM. Por outro lado, alguns registros nos EUA^77,78^, mostraram redução de mortalidade hospitalar associado com a administração de estatinas nas primeiras 24 horas do IAM. Além disso, benefícios na redução de insuficiência cardíaca foram descritos, tanto em terapia trombolítica com na angioplastia primária, também em pacientes que receberam estatinas nas primeiras 24 horas de admissão^79^.

No estudo ARMYDA-RECAPTURE^80^ foi mostrado que a administração de altas doses de atorvastatina, algumas horas antes da intervenção percutânea, se associou à menor elevação de marcadores de lesão miocárdica (troponina e CKMB) e melhor evolução livre de desfechos cardiovasculares nos primeiros 30 dias da intervenção, sugerindo que os efeitos pleiotrópicos e não as modificações lipídicas determinaram os principais benefícios da intervenção. Mais recentemente, estudo envolvendo rosuvastatina 40 mg pré intervenção percutânea mostrou melhor evolução em 12 meses para os principais desfechos cardiovasculares, em seguimento de 445 pacientes (9,8% vs. 20,5%, p=0,002)^81^. A sinvastatina constitui a mais frequente estatina utilizada nos hospitais durante a hospitalização por IAM, entretanto, como pró-droga, seus efeitos pleiotrópicos podem ser reduzidos pela concomitante prescrição de tienopiridínicos e outros medicamentos passíveis de interação farmacocinética, retardando a formação de metabólitos ativos.

O clopidogrel é uma pró-droga que sofre extensa metabolização hepática, por sucessivas isoenzimas do sistema microssomal CYP P450, até a formação do metabólito tiol ativo. Desta forma, fármacos que competem pelas isoenzimas CYP 1A2, 2C9, 2C19 e 3A4, são passiveis de interação com o clopidogrel. Recentemente examinamos os níveis séricos do clopidogrel 75 mg/dia e da atorvastatina 80 mg/dia, em coronarianos estáveis e observamos interação nas concentrações de ambos os fármacos, com aumento dos níveis de atorvastatina e redução dos níveis de clopidogrel, mas sem perda de benefícios na agregação plaquetária do clopidogrel ou nos efeitos hipolipemiantes da estatina. Entretanto, verificamos relação inversa entre o número de micropartículas plaquetárias e concentrações séricas do clopidogrel (AUC e CMax)^8^. Em outro estudo, com a rosuvastatina, observamos aumento nas concentrações da mesma após a introdução de clopidogrel (300 mg), mas muito menor efeito com a dose diária de 75 mg, sem interação da estatina nas concentrações do clopidogrel^59^. A melhora na função endotelial, examinada pela dilatação mediada pelo fluxo, foi expressiva nas primeiras 24 h, ao lado de efeitos sinérgicos na resposta antiagregante plaquetária e notável efeito hipolipemiante. Entretanto, perda parcial destes benefícios foi notada após uma semana da suspensão da estatina, a despeito do contínuo uso do tienopiridínico, com aumento de micropartículas plaquetárias^59^. Vistos em conjunto, os dados sugerem que a administração de rosuvastatina (20 mg) propicia benefícios precoces na doença coronariana aguda, pelo potencial sinergismo nas ações antitrombóticas e melhora da microcirculação coronariana.

Com relação ao ticagrelor, este antiplaquetário não tienopiridínico é o primeiro agente a bloquear reversivelmente e diretamente o receptor P2Y12, com mais rápido início de ação e maior grau de inibição plaquetária em comparação ao clopidogrel^82^. Além disso, benefícios inesperados na mortalidade, observados no estudo PLATO^83^, mas não com outro agente também de maior potência antiplaquetária em relação ao clopidogrel, o prasugrel no estudo TRITON-TIMI 38^84^, sugeriram que outros benefícios adicionais do ticagrelor possam ocorrer possam ocorrer por mecanismos além dos efeitos antiplaquetários^85-87^. De fato, recente manuscrito mostrou que o ticagrelor, mas não clopidogrel ou prasugrel, previne a contração muscular induzida pelo ADP^88^. Assim, é possível que a elevação das concentrações de adenosina com o ticagrelor seja o mecanismo responsável pela melhor evolução clínica, beneficiando a microcirculação e propiciando recuperação da função ventricular. Além disso, a reversibilidade da interação do ticagrelor com o receptor P2Y12 impediria aumento mais expressivo de sangramentos com reflexos na mortalidade cardiovascular.

O *myocardial stunning* descreve prolongada depressão da contratilidade miocárdica devido à isquemia, a despeito de adequada reperfusão^89^. Já o *myocardial hibernation* é usado para descrever uma forma mais persistente de disfunção contrátil reversível devido à doença arterial coronariana^90^. Os mecanismos deste último são menos compreendidos e nem sempre a perfusão está reduzida em áreas de miocárdio hibernado. Estudos recentes têm sugerido que a RNM-RT constitui o mais promissor método de imagem para detectar diferenças entre o miocárdio viável e infartado^91^. O método é automatizado, com alta reprodutibilidade e, embora os dados da literatura sejam ainda limitados, pequenos estudos têm mostrado valor prognóstico em relação a massa infartada obtida após intervenções^92,93^. A recuperação funcional do miocárdio isquêmico e estudos de *no-reflow*, complementarão as análises por RNM-RT de especial interesse nestes subgrupos de pacientes.

Vistos em conjunto, com este subprojeto pretendemos demonstrar que a introdução precoce e otimizada de rosuvastatina e ticagrelor determina benefícios imediatos na microcirculação coronariana, com reflexos no remodelamento ventricular nas primeiras semanas de IAM. A sinvastatina é amplamente utilizada no IAM, mas por ser pró-droga, poderá ser menos efetiva devido a necessária biotransformação hepática para produção de metabólitos ativos, cuja formação poderá ser influenciada pelo uso dos antiplaquetários que competem pelo mesmo sítio hepático de metabolização.

**2. Objetivos**

**2.1. Objetivos gerais**

- - 1. Comparar as quatro estratégias terapêuticas na função ventricular e quantidade de massa infartada, por meio de RNM-RT, precocemente (3-5 dias) e em dois períodos tardios (após quatro semanas e seis meses).

**2.2. Objetivos secundários**

- - 1. Avaliar a segurança e tolerabilidade dos tratamentos instituídos, por meio de estudos bioquímicos (modificações no perfil lipídico, enzimas musculares, hepáticas e biomarcadores cardíacos).
    2. Avaliar a taxa de sangramentos menores e maiores^80^.

1. **Métodos**

O estudo irá incluir pacientes com IAM com supra de ST tratados com trombolíticos nas primeiras 6 h de início dos sintomas. Serão incluídos pacientes de ambos os sexos com idade inferior a 75 anos. Serão excluídos pacientes com comorbidades que possam impedir a introdução dos fármacos ou a avaliação dos tratamentos, como hepatopatia ativa, sangramentos recentes, neoplasias, choque cardiogênico, conhecida intolerância aos fármacos do estudo, ou antecedentes pessoais que possam impedir uma adequada avaliação dos tratamentos (alcoolismo, dependência de drogas, doenças infecciosas ou reumatológicas crônicas, como AIDS, artrite reumatoide, LES, etc.).

O estudo será do tipo PROBE (*prospective, randomized, open label trial with blinded endpoints*) ou seja, aberto, aleatório e com análise cega de desfechos. O tamanho amostral foi calculado com base nos achados de RNM-RT em pacientes antes e após reperfusão coronária, mostrando que o método é capaz de identificar áreas reversíveis de miocárdio isquêmico^94^. Assim, foi estimado que os pacientes infartados possuíssem ao redor de 20% de área viável circundando o tecido necrótico que determinam hipocontratilidade segmentar, correspondendo a área de miocárdio viável. O tamanho amostral foi feito com base na diferença esperada entre as médias com 90% de poder da amostra para se detectar diferenças entre os grupos, simulando-se diferenças entre 10 a 20%, com risco alfa de 5%, e considerando-se em 30% o desvio-padrão da amostra, o tamanho amostral foi de 223 pacientes no pior cenário (Minn M. Soe and Kevin M. Sullivan, Emory University). Como não temos dados prévios desta intervenção e considerando o desenho fatorial 2x2, optamos por incluir o tamanho amostral de 300 pacientes (75 pacientes em cada braço do estudo). Os dados de RNM-RT serão analisados em laboratório central por dois experientes especialistas nestas modalidades de imagens. Coeficientes intra e inter os dois especialistas serão realizados a cada 1/3 de dados concluídos. O estudo será registrado como ensaio clínico antes de seu início e obedecerá as normas internacionais da boa prática clínica e harmonização de dados (GCP/ICH).

**Ressonância nuclear magnética e realce tardio**

A ressonância magnética será realizada com o objetivo de identificar e quantificar a área de necrose, isquemia e a zona de penumbra. Os exames serão realizados em equipamentos de alto campo (1,5 e 3,0 Tesla) com séries cine ressonância (imageamento rápido em imagens de equilíbrio em precessão - FIESTA), adquiridas durante apnéia de modo a garantir a reprodução de todo o ciclo cardíaco. Tais sequencias serão obtidas de forma a reproduzir as quatro cavidades cardíacas (cortes 4 câmaras) em projeções obliquas transversais e as câmaras ventriculares (cortes eixo curto) em projeções oblíquas sagitais. Os parâmetros básicos incluirão mínimo tempo de eco, tempo de repetição ao redor de 45 – 60 ms, ângulo de inclinação (*flipangle*) entre 30 e 60° em tantos cortes quantos sejam necessários para permitir a avaliação da espessura do miocárdio ao final de diástole e ao final de sístole, em todos as paredes ventriculares, de acordo com a divisão em 17 segmentos miocárdicos, conforme a classificação da *American Heart Association*^95^. A movimentação segmentar será quantificada de acordo com a avaliação objetiva em: normal, hipocinético, acinético e discinético. Adicionalmente, as imagens em eixo curto servirão para o cálculo do volume diastólico final, volume sistólico final, fração de ejeção global e massa miocárdica^96-104^. Em seguida, serão realizadas imagens de perfusão miocárdica. No caso da primeira avaliação o registro da opacificação do miocárdio será feita sem nenhum tipo de estresse. Nos exames posteriores, será feita acompanhada da injeção de 0,56 µg/kg de dipiridamol endovenoso, ou, se disponível, de 150 µg/kg/min de adenosina, seguida da administração de 1 mmol/kg de meio de contraste paramagnético (quelato de gadolínio), na velocidade de 3 ml/s. Tais imagens serão obtidas em múltiplas fases, de modo a permitir a quantificação da contrastação do miocárdio, que será feita de modo visual e quantitiativo, com o posicionamento de um cursor eletrônico, que medirá a intensidade de sinal ao longo da curva de tempo. As imagens serão registradas no eixo curto do coração, com obtenção de, no mínimo, três planos simultâneos, diferentes e paralelos. A presença de defeito de perfusão será definida como a área na qual não houver mudança significativa da intensidade de sinal após a injeção do meio de contraste paramagnético^104^.

A próxima série a ser obtida, será o mapeamento do tempo de relaxamento T1, utilizando imagem no meio do VE no eixo curto do coração obtida antes da injeção do meio de contraste paramagnético e repetida em intervalos de 5 min (somando seis pontos ao longo do tempo). Serão utilizadas sequências MOLLI (Modified Look Locker Inversion Recovery) ou sMOLLI (conforme já descritas na literatura – PMID 17896383 e PMID 21092085). Áreas com alta intensidade de sinal serão consideradas necrose/fibrose. Finalmente, entre 8-12 min após injeção do meio de contraste paramagnético, serão realizadas aquisições de realce tardio, com tempo de inversão segmentado e que terão o objetivo de demonstrar a fibrose ou necrose como zonas de elevada intensidade de sinal. Esta série objetiva quantificar a massa e percentual de necrose/fibrose miocárdica. Na avaliação inicial a presença de obstrução microvascular será aferida utilizando-se imagens de perfusão (nas quais este achado se apresenta mais frequente) e imagens de perfusão utilizando a técnica do realce tardio precoce (entre 2 e 5 min após a injeção IV do contraste, utilizando sequencia “single-shot” no eixo curto, que tem maior resolução espacial quando é feita a comparação com as imagens de perfusão) e tardio (após 10 min ou mais de injeção do contraste, utilizando sequências com segmentação de espaço K no eixo curto e nos eixos longos, com maior resolução espacial, nas quais a quantificação apresenta menor variabilidade de leitura – PMID 16915529, PMID 20348438, PMID 23021401). Além dos cálculos já expressos, a comparação das regiões de redução da contratilidade com as zonas de isquemia vistas `a perfusão e áreas de realce tardio definirão a diferença entre área isquêmica e zona de necrose. Estes resultados serão expressos em gramas de miocárdio e porcentual de miocárdio para permitir comparação entre os grupos^104^.

***SUBPROJETO 2***

**papel das células endoteliais progenitoras, micropartículas endoteliais E plaquetárias na evolução do INFARTO AGUDO DO MIOCÁRDIO**

**Resumo**

Fatores de risco clássicos da doença coronariana como diabetes, hipertensão, tabagismo e hipercolesterolemia, reduzem a mobilização de células endoteliais progenitoras (CEP) e estão associados à maior taxa de apoptose endotelial e risco trombótico. As estatinas podem aumentar a mobilização de CEP, particularmente após estímulos como intervenções percutâneas. Estudos prospectivos sugeriram que a taxa de CEP possui informação prognóstica, mas menos informação é disponível em relação ao papel das micropartículas (MP). Considerando-se que a oclusão coronariana está geralmente associada a um estado disfuncional do endotélio, a maior velocidade na recuperação functional e anatômica destas células pode se associar a melhora da microcirculação e recuperação do miocárdio isquêmico. Recente estudo de nosso grupo mostrou interação entre estatinas e clopidogrel nos níveis séricos destes fármacos, bem como nas taxas de CEP e MP plaquetárias e endoteliais. Assim, neste subprojeto iremos explorar possíveis benefícios da interação entre clopidogrel, ticagrelor, rosuvastatina e sinvastatina, na redução do miocárdio isquêmico que circunda o tecido necrótico nas primeiras semanas após IAM com supradesnível de segmento ST. Nossa hipótese é a de que o sinergismo entre estatina e antiplaquetários esteja associado a aumento na mobilização de CEP e redução do MP endoteliais e plaquetárias. Por meio da dilatação mediada pelo fluxo (DMF) e dos dados obtidos pela RNM-RT poderemos explorar estes aspectos. Com a utilização de CDs específicos, os elementos celulares descritos serão quantificados por citometria de fluxo, com metodologia estabelecida em nosso laboratório. A DMF seguirá as recentes recomendações da *European Society of Cardiology* e os pacientes serão aqueles incluídos no subprojeto 1, com desenho factorial 2x2.

1. **Fundamento da proposta**

O estado anatômico e funcional do endotélio vascular possui importantes implicações para a doença cardiovascular. Com o passar dos anos, foi descrita profunda mudança fenotípica do endotélio, que adquire características pró-inflamatórias, pró-oxidantes e pró-proliferativas^105^. A presença de fatores de risco clássicos impede a adequada reposição do endotélio senescente ou apoptótico, criando condições locais de maior risco de oclusões trombóticas^106^. Como a ausência anatômica do endotélio não é diagnosticada pelos estudos angiográficos convencionais, esta característica de risco cardiovascular parece subestimada. A presença de LDL oxidada, citocinas inflamatórias e baixa pressão de arrasto hemodinâmico constituem estímulos para apoptose endotelial, constituindo inicialmente respostas fisiológicas celulares, mas quando em excesso, identificam pacientes sob risco^107^. Dados mais recentes mostraram que a infusão de células CD 34+ (uma das características das células progenitoras endoteliais) se associou com melhora de perfusão miocárdica que pode limitar o comprometimento funcional cardíaco seis meses após IAM^108^. Em outro estudo, pacientes com maior número de células CD34+ tiveram melhor função cardíaca avaliada por RNM após três meses de evolução clínica^109^.

Trabalhos recentes de nosso grupo mostraram que a suspensão por uma semana de estatinas se acompanhou de aumento de MP plaquetárias, a despeito do uso contínuo de clopidogrel, em coronarianos^8^. Além disso, substancial melhora na função endotelial foi observada após 24 h da primeira dose de rosuvastatina 20 mg, também em coronarianos^59^. Em outros grupos populacionais, como pacientes portadores de HIV+ sem terapia retroviral, mostramos um desbalanço entre CEP e MP endoteliais, associadas à disfunção endotelial (DMF), sugerindo que a carga viral promova precoce desbalanço no *turnover* endotelial, propiciando um novo mecanismo para a elevada incidência de doença cardiovascular nestes pacientes^7^.

1. **Objetivos**
   1. **Objetivos gerais**
      1. Quantificar as CEP, MP endoteliais e plaquetárias na admissão e após 30 e 180 dias do IAM.
      2. Examinar possíveis diferenças nas quantificações de CEP e MP com base na estratégia terapêutica instituída (quatro braços do estudo).
   2. **Objetivos secundários**
      1. Examinar a função endotelial pela DMF (%) durante a hospitalização (3°-5° dias) e após 30 e 180 dias do IAM.
      2. Avaliar características da anatomia coronária, reperfusão, *blush* (microcirculação), *no-reflow* e outras características da cinecoronariografia disponíveis e examinar possíveis correlações com os dados da DMF, CEP e MP.
      3. Exame pela RNM-RT das características dos pacientes como *no-reflow* (previsto 10% da amostra) em relação a controles (relação casos e controles 1:3).
2. **Métodos**

As CEP e MP serão quantificadas por citometria de fluxo, com marcadores específicos, em amostras frescas de sangue obtidas nos períodos mencionados. A técnica para CPE foi padronizada em nosso laboratório e está descrita a seguir^7,8,59^. *Células endoteliais progenitoras.*

Serão colhidos aproximadamente 15 mL de sangue periférico dos pacientes em tubos com EDTA (para evitar coagulação), sendo acrescido Ficoll-Hypaque (Ficoll Paque Plus, GE Healthcare Bio-Sciences AB, Uppsala, Sweden) e centrifugado a 2000 rpm, 20-22ºC por 20 minutos, havendo separação de células mononucleares por gradiente de concentração. Posteriormente, as células obtidas são lavadas em solução isotônica (PBS). A viabilidade celular será analisada em hemocitômetro, após coloração de 10 uL das células com 90 uL de Azul de Tripan 60% (Sigma-Aldrich, MO, USA) por 5 minutos; em seguida as amostras são centrifugadas e imunomarcadas por 15 minutos à temperatura ambiente com os seguintes anticorpos: CD34 conjugado com isotiocianato de flouresceína – FITC (BD Biosciences, Franklin Lakes, NJ, USA), KDR conjugado com ficoeritrina - PE (R&D Systems, Minneapolis, USA) e CD133 conjugado com aloficocianina – APC (Miltenyi Biotec, Auburn, CA, USA). Como controles serão analisadas células marcadas com os isotipos IgG1 FITC (BD, Biosciences, Franklin Lakes, USA), IgG1 PE (R&D Systems, Minneapolis, MN) e IgG1-APC (Miltenyi Biotec, Auburn, USA). Imediatamente após este processo, é realizada a leitura em citômetro de fluxo (FACSCalibur – BD Biosciences, San Jose, USA) com análise feita pelo software Cell Quest Pro, antes e após o tratamento hipolipemiante. A identificação e quantificação de CPEé feita na região de linfócitos, pelas similaridades de morfologia, e os valores expressos em porcentagem (%) de CPE presentes nessa região

*Micropartículas Endoteliais e Plaquetárias*

Após coleta do sangue periférico dos pacientes em tubos de sangue contendo citrato, as amostras são centrifugadas a 4000 rpm, 20-22ºC por 10 minutos para a obtenção do plasma rico em plaquetas (PRP). O PRP é então centrifugado a 13000 rpm, 20-22ºC por 6 minutos, para a obtenção do plasma pobre em plaquetas (PPP). A seguir, 50 uL do PPP é duplamente marcado por 20 minutos, temperatura ambiente com o anti-CD42 conjugado com FITC e o anti-CD31 conjugado com PE (BD Biosciences, Franklin Lakes, NJ, USA). Segundo a literatura, o CD31 e o CD42 estão presentes em micropartículas plaquetárias. Além disso, as micropartículas endoteliais serão quantificadas utilizando o anti-CD51 conjugado com FITC associado ao anti-CD144 conjugado com PE (BD Biosciences, Franklin Lakes, NJ, USA). Como controles são utilizados os isotipos IgG1 FITC e IgG1 PE (BD Biosciences, Franklin Lakes, NJ, USA). As micropartículas serão quantificadas por microlitro de plasma pobre em plaquetas (PPP) que é injetado no citômetro.

Serão utilizados tubos TruCOUNT (Becton Dickinson) contendo uma quantidade conhecida de esferas (*beads*); estes tubos serão utilizados com o intuito de quantificar as micropartículas por microlitro de plasma pobre em plaquetas (PPP) injetado no citômetro.

A função endotelial será estimada pela DMF por equipamento de ultra-sonografia e transdutor linear de alta resolução. Iremos adotar as recomendações da ESC^111,112^ que preveem o registro dos últimos 30 s antes da liberação do manguito de pressão e registro por dois minutos após esta liberação. Todas as mensurações serão automatizadas. Os estudos serão realizados de maneira automatizada em aparelho de ultra-som VIVID 7 (GE Healthcare, USA), de alta resolução de imagem, com transdutor e software apropriados para estudos vasculares, que permitem até 1000 aquisições automatizadas do diâmetro da artéria braquial. Dois ecocardiografistas experientes realizarão o exame e a variabilidade intra e inter investigadores serão examinadas com 1/3 e 2/3 das pacientes incluídos no estudo.

***SUBPROJETO 3***

**Estudo DAS RESPOSTAS IMUNES E OXIDAÇÃO DA LDL na evolução do INFARTO AGUDO DO MIOCÁRDIO**

**Resumo**

A aterosclerose é hoje considerada uma doença vascular inflamatória, fortemente influenciada pela imunidade inata e adaptativa^113-115^. Estes estudos sugerem que alguns subtipos de linfócitos modulem a resposta fisiológica reparadora e outros agravem a sinalização inflamatória vascular relacionada aos eventos coronários trombóticos. Além disso, a imunidade adaptativa parece influenciar o remodelamento ventricular com base em modelos experimentais de infarto do miocárdio^116,117^. A participação da *vasa vasorum* na aterosclerose tem sido revista^118^ e parece desempenhar notável papel antiaterosclerótico, permitindo o efluxo de macrófagos da íntima vascular^119^. A presença de hipercolesterolemia parece reduzir a sinalização celular relacionada a este efluxo^119^, uma condição influenciada por estatina (efeito direto na hipercolesterolemia e indireto pleiotrópico). Além disso, foi sugerida interação do linfócito B1 com LDL oxidada na circulação e do linfócito B2 com a deposição de imunocomplexos na íntima vascular^120-126^. Em nossa instituição foi sintetizado um peptídio da apolipoproteína B (peptídio D), cuja porção é mais anfipática e sua detecção associada à maior grau de oxidação da lipoproteína.

Estudos de nosso grupo evidenciaram que a quantificação da resposta imune, por meio de anticorpos classe IgG para epitopos da apolipoproteína B incluindo respostas ao peptídio D desta apolipoproteína, está associada com a extensão da aterosclerose^29^, aos níveis pressóricos aumentados em hipertensos^28^, ao tratamento da hipertensão arterial^17^ e à instabilidade clínica nas síndromes isquêmicas agudas^33^. Além disso, análise do grau de oxidação de lipoproteínas pela varredura Z do laser constitui um dos objetivos do Instituto Nacional de Ciência e Tecnologia – Fluidos Complexos (INCT-FCx), do qual fazemos parte^127^. Por meio do coeficiente de difusibilidade do feixe de Laser, são obtidos dados quantitativos de alterações oxidativas das lipoproteinas, que se associam com alterações imunes dos anticorpos anti-LDL oxidadas^128^.

Assim, neste projeto pretendemos caracterizar e avaliar a participação da resposta imune pelo exame de subtipos de linfócitos (principalmente B1 e B2) na circulação, além de quantificarmos anticorpos da LDL oxidada e grau de oxidação pela varredura Z, buscando melhor compreensão do papel da imunidade adaptativa em relação ao remodelamento ventricular, necrose miocárdica, extensão da doença coronariana e desfechos cardiovasculares durante o seguimento de pacientes com IAM. Para este estudo, utilizaremos amostras dos 120 pacientes com IAM incluídos no subprojeto 1, mas com seguimento clínico ao longo de um ano. Assim, examinaremos os parâmetros mencionados na admissão e após 30, 180 e 360 dias após o IAM. Recentemente, novo e fascinante mecanismo a explicar as elevadas taxas de recorrência de eventos isquêmicos em pacientes com síndromes coronarianas agudas, principalmente ao longo do primeiro ano, foi descrito em resposta à necrose miocárdica^71^. O IAM determina grande mobilização de progenitoras medulares que estimula em órgãos linfóides, como o baço, maior liberação de linfócitos que acabam colonizando placas em desenvolvimento em outros sítios vasculares. Em outras palavras, o infarto do miocárdio acelera a aterosclerose e os desfechos recorrentes em coronarianos podem estar associados à desestabilização de placas devido ao excesso de linfócitos circulantes. Assim, este subprojeto, monitorando a produção de subtipos linfocitários, contribuirá para uma melhor compreensão do papel destas células na evolução da cardiopatia isquêmica.

1. **Fundamento da proposta**

A presença de lipoproteína de baixa densidade alterada por oxidação constitui um insulto vascular que deflagra uma pronta resposta reparativa, inicialmente mediada por nosso sistema imune inato. Na dependência das células envolvidas poderemos evoluir para resposta inflamatória de maior ou menor intensidade.

Entretanto, a imunidade adaptativa, uma resposta muito mais específica e evoluída da espécie humana, também possui um relevante papel na modulação da aterosclerose. Além disso, esta resposta imune pode determinar uma resposta reparadora, cicatricial do complexo processo inflamatório na íntima vascular, bem como envolvendo reações antígeno-anticorpos para a LDL oxidada (mediadas por linfócitos B), determinando sua depuração plasmática ou a deposição de imunocomplexos na íntima vascular.

**2. Objetivos**

**2.1. Objetivos gerais**

2.1.1. Caracterizar e quantificar subtipos de linfócitos B por citometria de fluxo na admissão e após 30, 180 e 360 dias do IAM.

2.1.2. Correlacionar os linfócitos B com a extensão de aterosclerose, grau de necrose miocárdica e remodelamento ventricular precoce e tardio.

- 1. **Objetivos secundários**
     1. Quantificar anticorpos da LDL oxidada durante a hospitalização (3°-5° dias) e após 30 e 180 dias do IAM.
     2. Examinar parâmetros de oxidação obtidos pela varredura Z do Laser.
     3. Verificar a associação entre os parâmetros de imunidade adaptativa obtidos e desfechos cardiovasculares durante o acompanhamento por um ano após IAM.

1. **Métodos**

Os subtipos de linfócitos B1 e B2 serão quantificados por CDs específicos, por citometria de fluxo.

*citometria para determinação de subtipos de linfócitos em humanos*

Serão coletados 10 mL de sangue de cada indivíduo em heparina. A este sangue será acrescentado igual volume de tampão fosfato (PBS). Esta mistura será gentilmente vertida em um gradiente de ficoll/hypaque (densidade 1,077). Para cada 3 ml de ficoll será vertido 10 mL da mistura. O sangue será submetido a centrifugação a 1300 rpm por 30 minutos. Serão formadas três fases no tubo sendo que as células mononucleares se concentram em forma de anel na interface entre plasma (na parte superior do tubo e hemácias e polimorfonucleares na base do tubo). O anel de células será recolhido, submetido a três etapas de lavagem com PBS e em seguida será determinada a viabilidade e concentração celular por contagem em câmara de Neubauer utilizando azul de trypan.

*Determinação das populações linfocitárias via expressão de marcadores específicos de superfície por citometria de fluxo*

As células (1 x 10^6^ células por tubo) serão marcadas com anticorpos monoclonais conjugados a fluorocromos para moléculas de superfície. Serão incubadas por 1 hora a 4ºC com 1 μL de anticorpos específicos, definindo as seguintes populações celulares: anti-CD19 conjugado a PE,anti-CD5 conjugado a PE-Cy7, anti-CD27 conjugado APC, anti-CD70, anti-CD20 e anti-CD43 para a população de células B1 totais; anti-CD19 conjugado a PE e anti-CD23 conjugado a FITC para a população de linfócitos B-2 (CD19^+^ CD23^+^) e anti-CD3, anti-CD4 e anti-CD8 para a população de linfócitos T. Após este período será realizada uma etapa de lavagem, e as células serão fixadas, adicionando-se 100 μL de PBS/BSA 1% e 400 μL de solução de paraformaldeído 1%, mantendo-as a 4ºC por 30 minutos. Após mais uma etapa de lavagem, as células serão ressuspensas em 1 mL de PBS e analisadas em citômetro de fluxo (FACSCANTO –BD ou ATTUNE –LIFE Technologies).

As análises serão feitas avaliando-se as porcentagens celulares obtidas nos dotplots (exemplo do esquema a seguir), de acordo com o programa do citometro e depois serão transformadas em número absoluto com base na contagem obtida nos leucogramas. Serão feitos experimentos para desenvolvimento de uma ou outra metodologia, de forma a caracterizar os subtipos de linfócitos humanos, para tornar estas quantificações consistentes e reprodutíveis.

Os anticorpos da LDL oxidada, bem como para o peptídio D da apolipoproteína B serão quantificados por ELISA.

*Detecção de anticorpos contra LDL oxidada (oxLDL)*

Para dosagem de auto-anticorpos contra oxLDL, será utilizado o método ELISA padronizado no laboratório do prof. Magnus Gidlund (Fernvik et al., 2004). Placas de 96 poços (Costar, EUA) serão sensibilizadas com 50 μL, na concentração de 7,5 μg/mL de oxLDL em tampão carbonato de sódio, 0,1 M, pH 9,4, durante 18h, a 4° C. Após 4 ciclos de lavagens com 100 µL de PBS, as placas serão bloqueadas com solução de gelatina a 1,0% (Gibco, EUA), em temperatura ambiente, por 24h. Em seqüência, as placas serão lavadas 4 vezes com PBS e os poços receberão, em triplicata, 50 μL das amostras dos diferentes grupos, diluídas 1:400 em PBS. Após 2 horas de incubação, as placas serão lavadas 4 vezes com 100 µL de PBS-T e incubadas com 50 μL de conjugado com peroxidase, por 1h, em temperatura ambiente. Será utilizado como conjugado imunoglobulina (Ig) G de cabra anti-IgG humana marcada com peroxidase (KLP, EUA) na diluição 1:1000. Posteriormente a mais 4 ciclos de lavagem, com PBS-T, o processo de revelação será realizado com a adição, em cada poço, de 75 µL de solução de TMB (250 µL de 3,3’5,5’-tetrametilbenzidina 6,5% em DMSO, 12 mL de tampão citrato 0,1M, pH 5,5 e 10 µL de H_2_O_2_ ). A reação será interrompida com a adição de 25 µL de ácido sulfúrico 2M (Merck, Alemanha). Os resultados serão obtidos por leitura espectrofotométrica a 450 nm em leitor de ELISA (GENIOS TECAN, Austria). Algumas imprecisões podem ocorrer no método de quantificação dos anticorpos por ELISA e para compensar a variação intra-placa nós utilizaremos em todas as placas como controle IgG purificada humana (purified human IgG – 10 mg/ml – Pierce Protein Research Products, Thermo Scientific, Rockford). Os títulos de anticorpos serão expressos como Índex de Reatividade (IR) para cada amostra e calculado da seguinte maneira: (Abs da amostra – Abs do Branco)/( Abs do controle de IgG - ABS do Branco).

A oxidação da LDL será estimada pelo coeficiente de difusibilidade ao Laser em amostras obtidas após ultracentrifugação de lipoproteínas por técnica padronizada no Instituto de Física da USP^125^.

Serão examinados 120 pacientes, 30 em cada braço do estudo, conforme as intervenções descritas no subprojeto 1.

***SUBPROJETO 4***

**ESTUDO DE METABOLITOS ENVOLVIDOS na evolução do INFARTO AGUDO DO MIOCÁRDIO**

**Resumo**

Metabolômica e lipidômica constituem novos ramos da ciência que propiciam, em conjunto, retrato bastante acurado e dinâmico de nossos sistemas biológicos integrados à bioinformática. As plataformas de cromatografia líquida/espectrometria de massa (LC-MS/MS) permitem que nucleotídeos, aminoácidos, ácidos orgânicos, carboidratos e lipides possam ser analisados. Assim, esta tecnologia permite a identificação de analitos associados com a doença cardiovascular e sua evolução, permitindo uma melhor compreensão das vias metabólitas envolvidas^129-132^. Modificações agudas relacionadas ao IAM em humanos foram recentemente descritas^133^, mas foram restritas a pequeno número de pacientes envolvidos em ablação por álcool para tratamento de cardiomiopatia hipertrófica. Este subprojeto examinará analitos de metabolômica associados `a evolução do IAM. Metabolômica e lipidômica têm sido propostas como importantes ferramentas para a melhor compreensão da sinalização inflamatória envolvida na aterosclerose^134-138^ e parecem adequadas ainda para uma melhor avaliação de terapias clínicas ou nutricionais propostas para esta doença. Neste subprojeto serão examinados os analitos detectados desde a fase aguda do IAM e ao longo de sua evolução, com ênfase à sua associação com parâmetros clínicos e dados obtidos pelos métodos de imagem.

**Fundamento da proposta:**

São pobremente conhecidos os determinantes da recuperação funcional e anatômica do infarto agudo do miocárdio^138,139^. A aplicação da metabolômica na análise evolutiva de grande número de indivíduos pode minimizar esperados viéses das amostras menores como comorbidades, idade, sexo e terapias concomitantes. Pela seleção específica de pacientes com infarto do miocárdio e levando-se em conta o seguimento do comportamento dos analitos em relação a parâmetros específicos, como função ventricular, quantidade de necrose miocárdica, terapias específicas e evolução clínica, novos biomarcadores poderão ser identificados. A comparação entre ticagrelor e clopidogrel permitirá melhor análise da adenosina, influenciando a necrose miocárdica e a fração de ejeção. Além disso, alguns marcadores de efeitos pleiotrópicos das estatinas poderão revelar diferenças entre rosuvastatina e sinvastatina.

**1. Objetivos:**

**1.1. Objetivos gerais:**

1.1.1. Quantificar analitos de metabolômica na admissão e após 30 e 180 dias após IAM e verificar sua associação com a necrose miocárdica e função ventricular.

**2. Objetivos secundários**

2.1. Verificar a correlação entre necrose miocárdica e títulos de adenosina.

2.2. Examinar a correlação entre metabólitos associados com a expressão de pequenas proteinas envolvidas na sinalização cellular dependente de estatinas e parâmetros de RNM-RT.

**3. Métodos**

**3.1.** Analitos de metabolômica serão quantificados por LC/MS-MS.

*Metabolômica*

Serão coletadas amostras de sangue periférico em frascos isentos de EDTA, dos mesmos sujeitos de pesquisa e na mesma ocasião. As amostras serão centrifugadas para precipitação da fase protéica, a ser descartada. O sobrenadante, que constitui o soro sanguíneo, será então transferido para *eppendorfs* limpos, que serão armazenados em *freezer* a 20ºC negativos até análise metabolômica. Uma amostra de controle de qualidade (QC) será preparada pela mistura de 5 μL de cada amostra individual e será usada para avaliar a estabilidade instrumental durante acquisição espectral de dados.

As amostras de soro serão analisadas por UPLC-MS/MS em um sistema cromatográfico de alta eficiência (UPLC Acquity, Waters Ltd., Elstree, U.K.) acoplado a um espectrômetro de massas híbrido de alta resolução (MicroMass, Waters MS Technologies Ltd., Manchester, U.K) com analisador do tipo Q-TOF, via ionização por electrospray, usando colunas de fase reversa Waters Acquity UPLC BEH C18 (1,8 μm, 2,1 x 100 mm) e fase normal (interação hidrofílica) Waters Acquity HILIC BEH (1,7 μm, 2,1 x 100 mm), operadas a 50°C e 40°C, respectivamente. Para a coluna C18: A = ácido fórmico a 0,1% em água, B = ácido fórmico a 0,1% em metanol. Eluição em gradiente com vazão de 0,4 mL/min: 0-2 min, 99,9% A:0,10% B; 6 min, 75% A:25% B; 10 min, 20% A:80% B, 12 min, 10% A:90% B, 21-23 min, 0,10% A:99,9% B, 24-26 min: 99,9% A:0,10% B. Para a coluna HILIC: A = 95% acetonitrila: 5% acetato de amônio a 200 mmol/L, contendo um total de 0.1% de ácido fórmico; B = 50% acetonitrila: 50% acetato de amônio a 20 mmol/L, contendo um total de 0,1% de ácido fórmico. Eluição em gradiente com vazão de 1,4 mL/min: 0-1 min, 99% A:1% B; 12 min, 100% B; 12,1-15 min, 99% A:1% B. Solvente forte é 5% acetonitrila e solvente fraco é 95% acetonitrila, ambos usados na lavagem da agulha do injetor automático.

Condições cromatográficas comuns a ambos os modos incluem temperatura do auto-amostrador (4°C) e volume de injeção (5 μL). Condições de partida para o espectrômetro de massas incluem: tensão do capilar de 3200 V (ionização positiva) ou 2400 V (ionização negativa), tensão do cone de 35 V, temperatura de dessolvatação de 350°C, temperatura da fonte de 120°C e vazões de gás de 25 L/h no cone e 900 L/h na dessolvatação. O Q-TOF será operado com ótica em V com velocidade de aquisição de dados de 0,2 s e interscan delay de 0,01 s. Uma solução de leucina encefalina (*m/z* 556,2771) a 200 pg/μL em 50% acetonitrila será usada como lockmass, sendo diretamente inserida no instrumento com vazão de 3 μL/min, via um sprayer auxiliar. Os dados serão coletados no modo centróide com faixa de varredura de 50 – 1000 *m/z*, com lockmass scans coletados a cada 15 s, sendo que uma média de 3 scans será usada para correção das massas.

Os dados brutos de UPLC-MS obtidos serão processados usando o software de acesso público XCMS (versão 1.24.1) que roda na plataforma R, gerando uma tabela de features (dados alinhados e normalizados), contendo *m/z*, tempos de retenção e intensidade de pico, para cada amostra. Os dados serão avaliados estatisticamente por métodos multivariados, incluindo PCA (principal components analysis), PLS-DA (projection to latent structure – discriminant analysis) e O-PLS-DA (PLS-DA ortogonal).^139-142^ A identidade dos metabólitos será obtida por pesquisa nos bancos de dados disponíveis: Human Metabolome Database (HMDB), Metlin, KEGG compound, and PubChem, e posteriormente confirmada por experimentos de fragmentação MS/MS e comparação com padrões autênticos. Os metabólitos que diferenciam as classes controle e teste serão associados a rotas metabólicas da base KEGG, com auxílio do MassTrix web server.

***SUBPROJETO 5***

**efeitos da mudança de flora intestinal na evolução do INFARTO AGUDO DO MIOCARDIO em pacientes DIABéTICOS E pré-diabéticos**

**Resumo**

Pacientes com síndromes coronarianas agudas possuem alta taxa de anormalidades do metabolismo de glicose, que incluem diabetes, pré-diabetes e hiperglicemia de estresse. Em pacientes com síndrome metabólica, estas alterações estão presentes em aproximadamente 75% da população^36^. Recentemente, notável contribuição do papel da mudança da microbiota intestinal em modelo experimental, mostrou a reversão de um fenótipo de resistência à insulina e obesidade^140^. Além disso, mostrou que a mudança de flora se acompanha de expressiva alteração em parâmetros inflamatórios, incluindo a modulação destas respostas por linfócitos T regulatórios do tecido visceral. É possível que a obesidade, microbiota e resistência à insulina modifiquem o balanço de subtipos de linfócitos e influenciem a evolução da aterosclerose, estabelecendo um elo entre a microbiota e a extensão da aterosclerose, bem como a evolução tardia de pacientes pré-diabéticos, diabeticos e obesos. Estudo experimental de nosso grupo mostrou que a aterosclerose experimental é profundamente influenciada pela presença de células inflamatórias peritoneais^10^. Neste subprojeto iremos examinar o papel da mudança de flora intestinal em parâmetros de resistência à insulina e metabolômica, bem como possíveis diferenças da quantificação de subtipos de linfócitos, quantificados na circulação, em pacientes com infarto agudo do miocárdio.

**1. Fundamento da proposta**

A aterosclerose mais extensa em diabéticos tipo 2 tem sido associada a múltiplos mecanismos, geralmente envolvendo maior atividade inflamatória, risco trombótico, disfunção endotelial, dislipidemia aterogênica (LDL pequenas e densas, hipertrigliceridemia, HDL-C baixo). Estes mecanismos de agravamento da doença aterosclerótico geralmente incidem em indivíduos obesos e sedentários, frequentemente classificados como portadores de glicemia de jejum alterada, tolerância diminuída à glicose ou classificados como portadores de síndrome metabólica. A presença desta última tem sido associada com maior mortalidade cardiovascular^141-151^. Entretanto, na presença de doença cardiovascular pré-existente, o prognóstico destes pacientes é ainda mais grave.

Atualmente estuda-se os mecanismos responsáveis pelo desenvolvimento de diabetes *mellitus* tipo 2 e obesidade, onde a microbiota intestinal é considerada como fator importante para a contribuição destes mecanismos.

Cani *et al*.^152^ propuseram que os lipopolissacarídeos (LPS) obtidos a partir da quebra de bactérias Gram-negativas que residem no intestino agem como desencadeadores do processo inflamatório. Continuamente produzidos por esse processo de lise celular, os LPS são fisiologicamente translocados por dentro dos capilares intestinais, transportados do intestino em direção aos tecidos pelas lipoproteínas, em resposta a uma dieta hiperlipidica.

O estudo de Creely *et al*^153^ revelou que em diabéticos tipo 2 há um aumento significativo de LPS no plasma quando comparados ao grupo placebo.

Os receptores *Toll-like* desempenham um papel fundamental na ativação da resposta imune em mamíferos. O receptor *Toll-like* (TLR) 4, é quem faz o reconhecimento dos LPS das bactérias Gram-negativas, e tem papel importante na inflamação e imunidade, sendo sua expressão relatada na maioria dos tecidos do corpo, inclusive os sensíveis à insulina. O TLR-4 é ativado por LPS e ácidos graxos saturados, que são indutores de resistência à insulina, TLR-4 pode ser um candidato para a participação no *cross-talk* entre os sinais inflamatórios e metabólicos^154^.

A ligação entre LPS e receptores TLR-4 produzem citocinas pró inflamatórias como TNF-α e IL-6^155^.

Os TLRs e as citocinas também possuem papel central na ligação da imunidade inata com a adaptativa, atuando sobre as células T e, particularmente, sobre as células dendríticas, mantendo um balanço fisiológico Th1 / Th2. A resposta Th1, caracterizada por excesso na produção de IFN- α, IL-2 e IL-12, esta associada com a eliminação de patógenos intracelulares e com doenças crônicas como diabetes e obesidade. É visto que os TLR-4 podem ser ativados por LPS, mas também por ácidos graxos saturados dietéticos induzindo a superestimulação de vias inflamatórias intracelulares, como JNK e NF-*kappa*β em adipócitos e macrófagos, relatados como sendo indutores de resistência à insulina e de aumento de adiposidade^156^.

O aumento de bactérias probióticas do tipo bifidobactérias, parece ser claramente eficaz na redução dos níveis de LPS, além de melhorar a função da barreira intestinal^157^.

Assim, neste subprojeto estaremos selecionando pacientes, na fase agudo do IAM, com diagnóstico de diabetes e pré-diabetes, com base nos critérios recentemente sugeridos pela *American Diabetes Association* (ADA) que se baseia nos níveis de hemoglobina glicada, o que constitui vantagem neste tipo de paciente pelas modificações agudas na glicemia e restrições ao teste de tolerância à glicose^158^. Nossa hipótese é a de que os pacientes diabéticos e pré-diabéticos possuam maior desbalanço em subtipos de linfócitos B associados com obesidade e características de sua flora intestinal. Nossa proposta é a de monitorarmos a flora intestinal destes pacientes por até seis meses e compararmos a microbiota e sua relação nos subtipos de linfócitos B, parâmetros de metabolômica e na evolução da cardiopatia isquêmica com base nos dados de ressonância nuclear magnética com realce tardio já mencionados em outro subprojeto. Serão também examinados parâmetros do metabolismo lipídico e sensibilidade à insulina. Nestes pacientes não serão feitas intervenções com pro-bióticos ou antibióticos, apenas monitoramento da flora e possível relação com os parâmetros mencionados.

**2. Objetivos**

**2.1. Objetivos gerais**

2.1.1. Comparar a composição da microbiota em pacientes pré-diabéticos, diabéticos e não diabéticos aos 30, 90 e 180 dias do IAM.

2.1.2. Examinar as subpopulações de linfócitos no sangue periférico aos 30 dias do infarto agudo do miocárdio e após 90 e 180 dias.

2.1.3. Comparar possíveis modificações nos analitos de metabolômica decorrentes do padrão de microbiota aos 30 dias, 90 e 180 dias após o infarto do miocárdio.

2.1.4. Comparar os achados de RNM-RT com o padrão de microbiota obtido.

**2.2. Objetivos secundários**

2.2.1. Verificar parâmetros de resistência à insulina (HOMA-IR) aos 30, 90 e 180 dias.

2.2.2. Comparar entre os grupos modificações de dados antropométricos aos 30, 180 e 180 dias.

**3. Métodos**

- 1. Linfócitos serão examinados por citometria de fluxo e CDs específicos.
  2. Estudos de metabolômica, que incluirá monitoramento do óxido de trimetilamina (TMAO)) serão realizados por LC/MS-MS.
  3. HbA1c será determinado por HPLC.

3.4. Microbiota será determinada por técnica KyberKompact Kyber plus, que permite a quantificação das principais bactérias patogênicas e benéficas da microbiota intestinal. O método diagnóstico Kyberplus oferece as informações a partir da matriz das fezes.

*População do estudo*

Serão incluídos neste subprojeto os pacientes que apresentarem valores de hemoglobina glicada (HbA1c) no primeiro dia de internação por IAM classificados como diabéticos, pré-diabéticos ou não diabéticos ou pré-diabéticos. Serão classificados como pré-diabéticos aqueles com valores de HbA1c entre 5,7% e 6,4%, como diabéticos os indivíduos com valores de HbA1c iguais ou superiores a 6,5%, e não-diabéticos ou pré-diabéticos (HbA1c <5,7%) conforme recomendação da *American Diabetes Association*  (ADA) de 2012. A determinação da HbA1c será feita por HPLC. O tratamento destes pacientes obedecerá as orientações gerais da ADA de 2012, incluindo mudanças no estilo de vida, de acordo com as recomendações do *National Cholesterol Education Program – Adult Treatment Panel* (NCEP-ATP) III^2^ e terapia farmacológica, quando necessária, de acordo com as recomendações.

Eventuais mudanças de flora intestinal serão analisadas para possíveis associações com alterações em parâmetros nutricionais, como medidas de prega cutânea, peso, índice de massa corpórea, circunferência abdominal, questionário de frequência alimentar, recordatório de 24h, bioimpedância e bioquímicos além dos estudos de metabolómica e de subtipos de linfócitos, já descritos.

**3.** **RESULTADOS ESPERADOS**

O projeto poderá identificar subtipos de linfócitos relacionados com a evolução da cardiopatia isquêmica. Nossa hipótese é a de um desequilíbrio em subtipos de linfócitos (maior presença de linfócitos B2 e menor de B1 e maior participação de linfócitos TCD4 em pacientes com fatores de risco clássicos, como diabetes, obesidade, sedentarismo, hipertensão e dislipidemias). Na presença do estímulo isquêmico decorrente do infarto agudo, maior proliferação destes linfócitos, também influenciados por maior apresentação de antígenos aos linfócitos T pelas células dendríticas, secundárias aos estímulos de constituintes inflamatórios e da LDL oxidada, determinarão maior massa infartada na fase aguda e maior progressão da cardiopatia isquêmica durante o seguimento dos pacientes, com respostas diferenciadas para os pacientes com maior exposição de linfócitos B. A persistência dos estímulos inflamatórios decorrentes do desequilíbrio dos linfócitos poderá afetar a evolução da cardiopatia isquêmica.

As estratégias de estatina e antiplaquetários (notadamente rosuvastatina e ticagrelor) poderão minimizar a perda muscular, pelo sinergismo na melhora da função endotelial nas primeiras horas do IAM. Entretanto, pelo fato da sinvastatina ser pró-droga e de sua possível interação com o clopidogrel, é possível que o benefício seja menor, por menor efeito pleiotrópico da estatina (antiinflamatótio, antitrombótico e na recuperação endotelial) e menor efeito do clopidogrel na microcirculação (possível diferença com base no esperado aumento da adenosina com o ticagrelor).

O padrão de flora intestinal constitui um novo elo entre as bactérias de nossa microbiota e a doença aterosclerótica, pois estudos recentes sugerem que a flora destes pacientes esteja associada com a produção de lipopolissacárides e subprodutos de constituintes alimentares de potencial impacto tanto na diferenciação de linfócitos como na instabilização de placas ateroscleróticas, além dos seus efeitos descritos na resistência à insulina.

Os analitos obtidos nos estudos de lipidômica e metabolômica poderão identificar vias metabólicas e mecanismos associados com a evolução da cardiopatia isquêmica, propiciando estratégias futuras, bem como maior compreensão e adequação das terapias propostas neste temático.

Estudos de anticorpos das classes IgM e IgG para a LDLoxidada permitirá importantes correlações com os linfócitos quantificados e evolução da cardiopatia isquêmica `a ressonância nuclear magnética.

O envolvimento de alunos de química analítica e da área biológica permitirá a formação de um núcleo de excelência nas áreas de metabolômica e imunologia, coordenado por docentes de grande experiência nestes campos e estimuladas pelos problemas contemplados no projeto.

O estudo integra quatro hospitais públicos de referência em São Paulo, Hospital São Paulo da UNIFESP, Instituto Dante Pazzanese de Cardiologia, Instituto do Coração da USP e Santa Casa de Misericórdia de São Paulo. Além disso, integra o Instituto de Química da USP, bem como do ICB-IV e Instituto de Física da USP aos Departamentos de Imunologia e Biofísica da UNIFESP.

Toda esta integração foi fruto de vários seminários e reuniões destinadas a estruturação do projeto, de forma a permitir o envolvimento e formação de alunos, menor alocação de recursos com terceiros e a perspectiva de alta produtividade destas colaborações, amparadas também na formação de núcleos centralizados para análises dos parâmetros de imagens e laboratoriais.

O estudo contempla todos os preceitos éticos da boa prática clínica e será registrado como ensaio clínico após sua aprovação ([www.clinicaltrials.g](http://www.clinicaltrials.g)ov).

**4. Desafios científicos e tecnológicos e os meios para superá-los**

A caracterização de subtipos de linfócitos em humanos foi um desafio no início do projeto, mas os docentes do Departamento de Imunologia da Unifesp rapidamente superaram estas dificuldades conforme os achados de nosso piloto.

A obtenção de amostras frescas de pacientes (como a quantificação de micropartículas e células progenitoras endoteliais e determinação de subtipos de linfócitos) ocorrerá apenas na Escola Paulista de Medicina e Instituto Dante Pazzanese de Cardiologia, pela proximidade das instituições o que reduzirá os custos do projeto, pois evitará o uso de transportadores (Courrier) de outras instituições. Os custos envolvidos em análises de subprojetos como metabolômica, oxidação de LDL, análises de função endotelial, entre outros foram revistos e adequados ao tamanho amostral necessário.

Os custos de imagem foram objeto de várias reuniões e foram minimizados, e terão custos SUS, ainda que surjam problemas técnicos com equipamentos das instituições públicas.

Medicamentos constituíam considerável parcela de custos do projeto e foram solicitados como doação de laboratórios, sem qualquer vínculo com o estudo, como acesso de dados, remuneração ou conflitos com investigadores.

Tendo em vista o tamanho amostral, foi discutida a participação das instituições de forma a obtermos o n necessário em quatro anos (média de sete pacientes incluídos/mês; sendo três da Unifesp, dois do IDPC e um do InCor e da Santa Casa), de forma a não conflitar com outros projetos em curso nestas instituições.

Será criada página na internet e prontuário eletrônico do estudo pelo Departamento de Informática da EPM/UNIFESP, incluindo banco de dados e senhas para segurança e armazenamento de dados.

Para adequação e monitoramento dos dados do estudo, uma comissão de três médicos (especialistas em imagens, cardiologia e epidemiologia/estatística) estará analisando os dados e informando a continuidade do projeto original ou sugerindo sua interrupção ao comitê central do estudo, a cada 1/3 dos pacientes incluídos).

1. **CRONOGRAMA DE EXECUÇÃO DO PROJETO**

|  | 2014 | 2015 | 2016 | 2017 | 2018 |
| --- | --- | --- | --- | --- | --- |
| Reuniões de abertura dos centros  Recrutamento dos pacientes  Reuniões de monitoramento de dados  Reuniões de grupos específicos*  Subprojeto 1  Subprojeto 2  Subprojeto 3  Subprojeto 4  Subprojeto 5 | **X**  **X**  **X**  **X**  **X**  **X**  **X**  **X** | **X**  **X**  **X**  **X**  **X**  **X**  **X**  **X** | **X**  **X**  **X**  **X**  **X**  **X**  **X**  **X** | **X**  **X**  **X**  **X**  **X**  **X**  **X**  **X** | **X**  **X**  **X**  **X**  **X**  **X**  **X** |

*Comitê envolvendo pesquisadores associados e comitês específicos de RNM, imunologia e lipidômica/metabolômica.

**6. pesquisadores ASSOCIADOs**

*Imunidade Adaptativa*

Prof. Dr. José Daniel Lopes – Departamento de Imunologia da EPM/UNIFESP

Prof. Dr. Mario Mariano – Departamento de Imunologia da EPM/UNIFESP

Profa. Dra.Ieda Longo Maugeri – Departamento de Imunologia da EPM/UNIFESP

*Ressonância Nuclear Magnética*

Dr. Gilberto Szarf – EPM/UNIFESP e Laboratório Fleury

Dr. Ibrahim Maschiarelli Pinto – IDPC e Laboratório Fleury

*Metabolômica*

Profa. Dra. Marina F. M. Tavares – Instituto de Química da USP

Dra. Aline Klassen – Instituto de Química da USP

*Oxidação de lipoproteínas e síntese de peptídios*

Prof. Dra. Maria Cristina O Izar – Disciplina de Cardiologia EPM/UNIFESP

Prof. Dr. Magnus Gidlund – ICB IV – USP

Dra. Andrea Monteiro – ICB IV - USP

Prof. Dr. Antonio Martins Figueiredo – Instituto de Física da USP

Prof. Dr. Luiz A Juliano – Departamento de Biofísica da UNIFESP

*Estudos hemodinâmicos*

Dra. Claudia Maria Rodrigues Alves - Disciplina de Cardiologia EPM/UNIFESP

Dr. Alexandre Abizaid – IDPC

Dr. Adriano Caixeta - Disciplina de Cardiologia EPM/UNIFESP

*Protocolo clínico/Unidade coronária*

Prof. Dr. Antonio C C Carvalho - Disciplina de Cardiologia EPM/UNIFESP

Dr. Rui Fernando Ramos – IDPC

Prof. Dr. José Carlos Nicolau –InCor/USP

Prof. Dr. Roberto Franken – Santa Casa de Misericórdia de São Paulo

*Comitê independente de monitorização dos dados do estudo*

Dr. Otavio Bewander – cardiologista/epidemiologista – HCOR/IDPC

Dr. Carlos Rochitte –imagem/cardiologia - InCor/USP

Prof. Dr. Luiz Antonio Machado Cesar – coronária - InCor/USP

*Comitê de informática – Banco de dados e prontuário eletrônico*

Orlando Lima Cardoso – Diretor de Tecnologia da Informação do Hospital São Paulo

**Referências:**

1. Fonseca FA, Paiva TB, Silva EG, *et al*. Atherosclerosis. 1998;139:237-42.
2. Fonseca FA, Izar MC, Fuster V, *et al*. Atherosclerosis. 2001;154:61-9.
3. Silva EP, Fonseca FA, Ihara SS, *et al*. J Cardiovasc Pharmacol. 2002;39:389-95.
4. Fonseca FA, Ihara SS, Izar MC, *et al*. Clin Exp Pharmacol Physiol. 2003;30:779-85.
5. Ferreira WP, Bertolami MC, Santos SN, *et al*. Pediatr Cardiol. 2007;28:8-13.
6. Monteiro CM, Pinheiro LF, Izar MC, *et al*. Braz J Med Biol Res. 2010;43:297-302.
7. da Silva EF, Fonseca FA, França CN, *et al*. AIDS. 2011;25:1595-601.
8. [França CN](https://www.ncbi.nlm.nih.gov/pubmed?term=Fran%C3%A7a%20CN%5BAuthor%5D&cauthor=true&cauthor_uid=22214900), [Pinheiro LF](https://www.ncbi.nlm.nih.gov/pubmed?term=Pinheiro%20LF%5BAuthor%5D&cauthor=true&cauthor_uid=22214900), [Izar MC](https://www.ncbi.nlm.nih.gov/pubmed?term=Izar%20MC%5BAuthor%5D&cauthor=true&cauthor_uid=22214900), *et al*. Circ J. 2012;76:729-36.
9. Pomaro DR, Ihara SS, Pinto LE, *et al*. J Cardiovasc Pharmacol. 2005;45:295-300.
10. Relvas WG, Izar MC, Segreto HR, *et al*. J Atheroscler Thromb. 2010;17:378-85.
11. Helfenstein T, Fonseca FA, Ihara SS, *et al*. Int J Exp Pathol. 2011;92:40-9.
12. [Feio CA, Izar MC, Ihara SS, *et al*.](http://www.ncbi.nlm.nih.gov/pubmed/22139433) J Atheroscler Thromb. 2011. [Epub ahead of print]
13. Albert MA, Glynn RJ, Fonseca FA, *et al*. Am Heart J. 2011;162:106-14.
14. de Lima Sanches P, de Mello MT, Elias N, *et al*. Hypertens Res. 2011;34:232-8.
15. Fonseca FA, França CN, Póvoa RM, Izar MC. Rev Neurol. 2010;51:551-60.
16. Ridker PM, MacFadyen JG, Fonseca FA, *et al*. Circ Cardiovasc Qual Outcomes. 2009;2:616-23.
17. Brandão SA, Izar MC, Fischer SM, *et al*. Am J Hypertens. 2010;23:208-14.
18. Fonseca FA, Izar MC. Expert Rev Cardiovasc Ther. 2009;7:1041-56.
19. Santos AO, Fonseca FA, Fischer SM, *et* al. Clin Chim Acta. 2009;406:113-8.
20. Glynn RJ, Danielson E, Fonseca FA, *et al*. N Engl J Med. 2009;360:1851-61.
21. Ridker PM, Danielson E, Fonseca FA, *et* al. Lancet. 2009;373:1175-82.
22. Ridker PM, Danielson E, Fonseca FA, *et al*. N Engl J Med. 2008;359:2195-207.
23. Ridker PM, Fonseca FA, Genest J, *et al*. Am J Cardiol. 2007;100:1659-64.
24. Ramos SC, Fonseca FA, Kasmas SH, *et al*. Nutr J. 2011;10:80.
25. Izar MC, Tegani DM, Kasmas SH, Fonseca FA. Genes Nutr. 2011;6:17-26.
26. Fonseca HA, Izar MC, Bianco HT, Fonseca FA. J Atheroscler Thromb. 2010;17:888.
27. Kasmas SH, Izar MC, França CN, *et al*. Braz J Med Biol Res 2012 (in press).
28. da Fonseca HA, Fonseca FA, Monteiro AM, *et al*. Int J Cardiol. 2012;157:131-3.
29. Izar MC, Fonseca HA, *et al.* Diab Vasc Dis Res. 2012. [Epub ahead of print].
30. [Colossimo AP, Costa Fde A, Riera AR, *et al.*](http://www.ncbi.nlm.nih.gov/pubmed/21845342) Arq Bras Cardiol. 2011;97:225-31.
31. Costa F de A, Bombig MT, de Lima VC, *et al*. Int J Cardiol. 2011;151:374-5.
32. Marui FR, Bombig MT, Francisco YA, *et al*. Arq Bras Cardiol. 2010;95:536-40.
33. Santos MA, Costa Fde A, Travessa AF, *et al*. Arq Bras Cardiol. 2010;94:620-4.
34. Schwartz GG, Olsson AG, Ballantyne CM, *et al*. Am Heart J. 2009;158:896-901.
35. Brollo L, Bombig MT, Mazzaro CL, *et al*. Arq Bras Cardiol. 2009;92:351-5.
36. Monteiro CM, Oliveira L, Izar MC, *et al*. Arq Bras Cardiol. 2009;92:89-99.
37. da Costa W, Riera AR, Costa Fde A, *et al*. J Electrocardiol. 2008;41:724-9.
38. Izar MC, Helfenstein T, Ihara SS,*et al*. Atherosclerosis. 2009;204:165-70.
39. Mazzaro C do L, Costa F de A, Bombig MT, *et al*. Arq Bras Cardiol. 2008;90:227-31.
40. Sposito AC, Caramelli B, Fonseca FA, *et al*. Arq Bras Cardiol. 2007;88 Suppl 1:2-19.
41. Teixeira M, Kasinski N, Izar MC, *et al*. Arq Bras Cardiol. 2006;87:3-11.
42. Middleton A, Binbrek AS, Fonseca FA, *et al*. Curr Med Res Op. 2006;22:1181-91.
43. Fonseca FA, Izar MC, Silva MA, *et al.* Evid Based Cardiovasc Med. 2006;10:96-100.
44. Mendes GA, Martinez TL, Izar MC, *et al*. Arq Bras Cardiol. 2006;86:361-5.
45. Back Giuliano I, Caramelli B, Pellanda L, *et al*. Arq Bras Cardiol. 2005;85:4-36.
46. Fonseca FA. Arq Bras Cardiol. 2005 Oct;85 Suppl 5:9-14.
47. Fonseca FA, Ruiz A, Cardona-Muñoz EG, *et al* Curr Med Res Opin. 2005;21:1307-15.
48. Helfenstein T, Fonseca FA, Relvas WG, *et al*. Clin Chim Acta. 2005;355:165-72.
49. Relvas WG, Izar MC, Helfenstein T, *et al*. Atherosclerosis. 2005;178:101-5.
50. Fonseca FA, Izar MC. Arq Bras Cardiol. 2004;83:371-2.
51. Elias MC, Bolívar MS, Fonseca FA, *et al*. Arq Bras Cardiol. 2004;82:143-6.
52. Feio CM, Fonseca FA, Rego SS, *et al*. Arq Bras Cardiol. 2003;81:596-9.
53. Bricarello LP, Kasinski N, Bertolami MC, *et al*. Nutrition. 2004;20:200-4.
54. Izar MC, Fonseca FA, Ihara SS, *et al*. [.](http://www.ncbi.nlm.nih.gov/pubmed/12754559)Arq Bras Cardiol. 2003;80:379-95.
55. Santos RD, Spósito AC, dos Santos JE, *et al*. Arq Bras Cardiol. 2000;75:289-302.
56. Fonseca FA, Novazzi JP, Cendoroglo MS, *et al*. Arq Bras Cardiol. 1996;66:33-5.
57. Novazzi JP, Fonseca FA, Feres MC, *et al*. Arq Bras Cardiol. 1994;62:395-8.
58. de Carvalho VB, Fonseca FA, *et al*. Arq Bras Cardiol. 1991;57:41-5.
59. [Pinheiro LF](https://www.ncbi.nlm.nih.gov/pubmed?term=Pinheiro%20LF%5BAuthor%5D&cauthor=true&cauthor_uid=22569318), [França CN](https://www.ncbi.nlm.nih.gov/pubmed?term=Fran%C3%A7a%20CN%5BAuthor%5D&cauthor=true&cauthor_uid=22569318), [Izar MC](https://www.ncbi.nlm.nih.gov/pubmed?term=Izar%20MC%5BAuthor%5D&cauthor=true&cauthor_uid=22569318), *et al.* Int J Cardiol 2012;158:125-8.
60. Pereira IA, Borba EF, 2008. Swiss Med. Wkly 2008;138:534–9.
61. Moore KJ, Tabas I. Cell 2011;145:341–55.
62. Weber C, *et al.* J Clin Invest 2011;121:2898–910.
63. Vitale G, *et al*. Mol. Immunol. 2010;48:1–8.
64. Miller YI, *et al*. Circ Res 2011;108:235-48.
65. Galkina E.*et al*. J Exp Med 2006;203:1273–82.
66. Ponnuswamy P, Van Vré EA, Mallat Z, Tedgui A. Vasc Pharmacol 2012;56:193-203.
67. van Gils JM, Derby MC, Fernandes LR, *et al*. Nat Immunol 2012;13:136-43.
68. Campbell KA, Lipinski MJ, Doran AC, *et al*. Circ Res 2012;110:889-900.
69. Gerszten RE, Tager AM. N Engl J Med 2012;366:1734-6.
70. Goodchild TT, Robinson KA PHD, *et al*. JACC cardiovasc Interv 2009; 2:1005–16.
71. Dutta P, Courties G, Wei Y, *et al*. Nature 2012 (Epub ahead of print).
72. Moreira FT, Ramos SC, Monteiro AM, et al. Life Sci 2014;98:83-7
73. Hilgendorf I, Theurl I, Gerhardt LMS, et al. Circulation 2014;129:1677-87.
74. Kyaw T, Toy C, Krishnamurth S. Circ Res 2011;109:830-40.
75. [Schwartz GG](http://www.ncbi.nlm.nih.gov/pubmed?term=Schwartz%20GG%5BAuthor%5D&cauthor=true&cauthor_uid=11277825), [Olsson AG](http://www.ncbi.nlm.nih.gov/pubmed?term=Olsson%20AG%5BAuthor%5D&cauthor=true&cauthor_uid=11277825), [Ezekowitz MD](http://www.ncbi.nlm.nih.gov/pubmed?term=Ezekowitz%20MD%5BAuthor%5D&cauthor=true&cauthor_uid=11277825), *et al*. JAMA. 2001;285:1711-8.
76. [Cannon CP](http://www.ncbi.nlm.nih.gov/pubmed?term=Cannon%20CP%5BAuthor%5D&cauthor=true&cauthor_uid=15007110), [Braunwald E](http://www.ncbi.nlm.nih.gov/pubmed?term=Braunwald%20E%5BAuthor%5D&cauthor=true&cauthor_uid=15007110), [McCabe CH](http://www.ncbi.nlm.nih.gov/pubmed?term=McCabe%20CH%5BAuthor%5D&cauthor=true&cauthor_uid=15007110), *et al*. N Engl J Med. 2004;350:1495-504.
77. [Spencer FA](http://www.ncbi.nlm.nih.gov/pubmed?term=Spencer%20FA%5BAuthor%5D&cauthor=true&cauthor_uid=15505131), [Fonarow GC](http://www.ncbi.nlm.nih.gov/pubmed?term=Fonarow%20GC%5BAuthor%5D&cauthor=true&cauthor_uid=15505131), [Frederick PD](http://www.ncbi.nlm.nih.gov/pubmed?term=Frederick%20PD%5BAuthor%5D&cauthor=true&cauthor_uid=15505131), *et al*. Arch Intern Med. 2004;164:2162-8.
78. [Fonarow GC](http://www.ncbi.nlm.nih.gov/pubmed?term=Fonarow%20GC%5BAuthor%5D&cauthor=true&cauthor_uid=16125480), [Wright RS](http://www.ncbi.nlm.nih.gov/pubmed?term=Wright%20RS%5BAuthor%5D&cauthor=true&cauthor_uid=16125480), [Spencer FA](http://www.ncbi.nlm.nih.gov/pubmed?term=Spencer%20FA%5BAuthor%5D&cauthor=true&cauthor_uid=16125480), *et al.* Am J Cardiol. 2005;96:611-6.
79. [Wright RS](http://www.ncbi.nlm.nih.gov/pubmed?term=Wright%20RS%5BAuthor%5D&cauthor=true&cauthor_uid=15975672), [Bybee K](http://www.ncbi.nlm.nih.gov/pubmed?term=Bybee%20K%5BAuthor%5D&cauthor=true&cauthor_uid=15975672), [Miller WL](http://www.ncbi.nlm.nih.gov/pubmed?term=Miller%20WL%5BAuthor%5D&cauthor=true&cauthor_uid=15975672), *et al*. Int J Cardiol. 2006;108:314-9.
80. [Di Sciascio G](http://www.ncbi.nlm.nih.gov/pubmed?term=Di%20Sciascio%20G%5BAuthor%5D&cauthor=true&cauthor_uid=19643320), [Patti G](http://www.ncbi.nlm.nih.gov/pubmed?term=Patti%20G%5BAuthor%5D&cauthor=true&cauthor_uid=19643320), [Pasceri V](http://www.ncbi.nlm.nih.gov/pubmed?term=Pasceri%20V%5BAuthor%5D&cauthor=true&cauthor_uid=19643320), *et al*. J Am Coll Cardiol. 2009;54:558-65.
81. [Yun KH](http://www.ncbi.nlm.nih.gov/pubmed?term=Yun%20KH%5BAuthor%5D&cauthor=true&cauthor_uid=20471117), [Oh SK](http://www.ncbi.nlm.nih.gov/pubmed?term=Oh%20SK%5BAuthor%5D&cauthor=true&cauthor_uid=20471117), [Rhee SJ](http://www.ncbi.nlm.nih.gov/pubmed?term=Rhee%20SJ%5BAuthor%5D&cauthor=true&cauthor_uid=20471117), *et al*. Int J Cardiol. 2011;146:68-72.
82. [Gurbel PA](https://www.ncbi.nlm.nih.gov/pubmed?term=Gurbel%20PA%5BAuthor%5D&cauthor=true&cauthor_uid=19923168), [Bliden KP](https://www.ncbi.nlm.nih.gov/pubmed?term=Bliden%20KP%5BAuthor%5D&cauthor=true&cauthor_uid=19923168), [Butler K](https://www.ncbi.nlm.nih.gov/pubmed?term=Butler%20K%5BAuthor%5D&cauthor=true&cauthor_uid=19923168), *et al.* Circulation. 2009;120:2577-85.
83. [Wallentin L](https://www.ncbi.nlm.nih.gov/pubmed?term=Wallentin%20L%5BAuthor%5D&cauthor=true&cauthor_uid=19717846), [Becker RC](https://www.ncbi.nlm.nih.gov/pubmed?term=Becker%20RC%5BAuthor%5D&cauthor=true&cauthor_uid=19717846), [Budaj A](https://www.ncbi.nlm.nih.gov/pubmed?term=Budaj%20A%5BAuthor%5D&cauthor=true&cauthor_uid=19717846), *et al.* N Engl J Med. 2009;361:1045-57.
84. Wiviott SD, Braunwald E, McCabe CH, *et al*. N Engl J Med. 2007;357:2001-15.
85. [Serebruany VL](https://www.ncbi.nlm.nih.gov/pubmed?term=Serebruany%20VL%5BAuthor%5D&cauthor=true&cauthor_uid=21212672). Cardiology. 2010;117:231-3.
86. [Serebruany VL](https://www.ncbi.nlm.nih.gov/pubmed?term=Serebruany%20VL%5BAuthor%5D&cauthor=true&cauthor_uid=20024505). Thromb Haemost. 2010;103:259-61.
87. [Serebruany VL](https://www.ncbi.nlm.nih.gov/pubmed?term=Serebruany%20VL%5BAuthor%5D&cauthor=true&cauthor_uid=20007979), [Atar D](https://www.ncbi.nlm.nih.gov/pubmed?term=Atar%20D%5BAuthor%5D&cauthor=true&cauthor_uid=20007979). Eur Heart J. 2010;31:764-7.
88. [Grzesk G](https://www.ncbi.nlm.nih.gov/pubmed?term=Grzesk%20G%5BAuthor%5D&cauthor=true&cauthor_uid=22265722), [Kozinski M](https://www.ncbi.nlm.nih.gov/pubmed?term=Kozinski%20M%5BAuthor%5D&cauthor=true&cauthor_uid=22265722), [Navarese EP](https://www.ncbi.nlm.nih.gov/pubmed?term=Navarese%20EP%5BAuthor%5D&cauthor=true&cauthor_uid=22265722), *et al*. Thromb Res. 2012. [Epub ahead of print].
89. [Braunwald E](https://www.ncbi.nlm.nih.gov/pubmed?term=Braunwald%20E%5BAuthor%5D&cauthor=true&cauthor_uid=6754130), [Kloner RA](https://www.ncbi.nlm.nih.gov/pubmed?term=Kloner%20RA%5BAuthor%5D&cauthor=true&cauthor_uid=6754130). Circulation. 1982;66:1146-9.
90. [Rahimtoola SH](https://www.ncbi.nlm.nih.gov/pubmed?term=Rahimtoola%20SH%5BAuthor%5D&cauthor=true&cauthor_uid=2783527). Am Heart J. 1989;117:211-21.
91. [Saraste A](https://www.ncbi.nlm.nih.gov/pubmed?term=Saraste%20A%5BAuthor%5D&cauthor=true&cauthor_uid=18242487), [Nekolla S](https://www.ncbi.nlm.nih.gov/pubmed?term=Nekolla%20S%5BAuthor%5D&cauthor=true&cauthor_uid=18242487), [Schwaiger M](https://www.ncbi.nlm.nih.gov/pubmed?term=Schwaiger%20M%5BAuthor%5D&cauthor=true&cauthor_uid=18242487). J Nucl Cardiol. 2008;15:105-17.
92. [Roes SD](https://www.ncbi.nlm.nih.gov/pubmed?term=Roes%20SD%5BAuthor%5D&cauthor=true&cauthor_uid=17826372), [Kelle S](https://www.ncbi.nlm.nih.gov/pubmed?term=Kelle%20S%5BAuthor%5D&cauthor=true&cauthor_uid=17826372), [Kaandorp TA](https://www.ncbi.nlm.nih.gov/pubmed?term=Kaandorp%20TA%5BAuthor%5D&cauthor=true&cauthor_uid=17826372), *et al.* Am J Cardiol. 2007;100:930-6.
93. [Bello D](https://www.ncbi.nlm.nih.gov/pubmed?term=Bello%20D%5BAuthor%5D&cauthor=true&cauthor_uid=15808771), [Fieno DS](https://www.ncbi.nlm.nih.gov/pubmed?term=Fieno%20DS%5BAuthor%5D&cauthor=true&cauthor_uid=15808771), [Kim RJ](https://www.ncbi.nlm.nih.gov/pubmed?term=Kim%20RJ%5BAuthor%5D&cauthor=true&cauthor_uid=15808771), *et al*. J Am Coll Cardiol. 2005;45:1104-8.
94. [Kim RJ](https://www.ncbi.nlm.nih.gov/pubmed?term=Kim%20RJ%5BAuthor%5D&cauthor=true&cauthor_uid=11078769), [Wu E](https://www.ncbi.nlm.nih.gov/pubmed?term=Wu%20E%5BAuthor%5D&cauthor=true&cauthor_uid=11078769), [Rafael A](https://www.ncbi.nlm.nih.gov/pubmed?term=Rafael%20A%5BAuthor%5D&cauthor=true&cauthor_uid=11078769), *et al*. N Engl J Med. 2000;343:1445-53.
95. Cerqueira MD, Weissman NJ, Dilsizian V, *et al*. Circulation 2002;105:539-42.
96. Kramer CM. Cardiol Clin 1998;16:267-76.
97. Gudmundsson P, Winter R, Dencker M, *et al*. Clin Physiol Funct Imag 2006;26:32-8.
98. Stork A, Muellerleile K, Bansmann PM, *et al*. Eur Radiol 2007;17:610-7.
99. KnoppMV, Schoenberg SO, Rehm C, *et al*. Invest Radiol 2002;37:706-15.

100. McCrohon JA, Moon JC, Prasad SK, *et al*. Circulation 2003;108:54-9

101. Vohringer M, Mahrholdt H, Yilmaz A, Sechtem U. Herz 2007;32:129-37.

102. Mahrholdt H, Wagner A, Judd RM, *et al*. Eur Heart J 2005;26:1461-74.

103. Weinsaft JW, Klem I,Judd RM. Cardiol Clin 2007;25:35-56.

104. Sparrow P, Messroghli DR, Reid S, *et al*. Am J Radiol 2006;187:W630–W635.

105. [Camici GG](http://www.ncbi.nlm.nih.gov/pubmed?term=Camici%20GG%5BAuthor%5D&cauthor=true&cauthor_uid=19434051), [Sudano I](http://www.ncbi.nlm.nih.gov/pubmed?term=Sudano%20I%5BAuthor%5D&cauthor=true&cauthor_uid=19434051), [Noll G](http://www.ncbi.nlm.nih.gov/pubmed?term=Noll%20G%5BAuthor%5D&cauthor=true&cauthor_uid=19434051), *et al*. Curr Opin Nephrol Hypertens. 2009;18:134-7.

106. [Werner N](http://www.ncbi.nlm.nih.gov/pubmed?term=Werner%20N%5BAuthor%5D&cauthor=true&cauthor_uid=17453672), [Nickenig G](http://www.ncbi.nlm.nih.gov/pubmed?term=Nickenig%20G%5BAuthor%5D&cauthor=true&cauthor_uid=17453672). Ann Med. 2007;39:82-90.

107. [Boulanger CM](http://www.ncbi.nlm.nih.gov/pubmed?term=Boulanger%20CM%5BAuthor%5D&cauthor=true&cauthor_uid=16801490), [Amabile N](http://www.ncbi.nlm.nih.gov/pubmed?term=Amabile%20N%5BAuthor%5D&cauthor=true&cauthor_uid=16801490), [Tedgui A](http://www.ncbi.nlm.nih.gov/pubmed?term=Tedgui%20A%5BAuthor%5D&cauthor=true&cauthor_uid=16801490). Hypertension. 2006;48:180-6.

108. [Quyyumi AA](http://www.ncbi.nlm.nih.gov/pubmed?term=Quyyumi%20AA%5BAuthor%5D&cauthor=true&cauthor_uid=21167340), [Waller EK](http://www.ncbi.nlm.nih.gov/pubmed?term=Waller%20EK%5BAuthor%5D&cauthor=true&cauthor_uid=21167340), [Murrow J](http://www.ncbi.nlm.nih.gov/pubmed?term=Murrow%20J%5BAuthor%5D&cauthor=true&cauthor_uid=21167340), *et al*. Am Heart J. 2011;161:98-105.

109. Geisler T, Fekecs L, Wurster T, *et al*. Eur J Radiol. 2012;81:e486-90.

110. Sibbing D, Braun S, Morath T, *et al*. J Am Coll Cardiol 2009; 53: 849-56.

111. [Gori T](http://www.ncbi.nlm.nih.gov/pubmed?term=Gori%20T%5BAuthor%5D&cauthor=true&cauthor_uid=21920964), [Muxel S](http://www.ncbi.nlm.nih.gov/pubmed?term=Muxel%20S%5BAuthor%5D&cauthor=true&cauthor_uid=21920964), [Damaske A](http://www.ncbi.nlm.nih.gov/pubmed?term=Damaske%20A%5BAuthor%5D&cauthor=true&cauthor_uid=21920964), *et al*. Eur Heart J. 2012;33:363-71.

112. [Spiro JR](http://www.ncbi.nlm.nih.gov/pubmed?term=Spiro%20JR%5BAuthor%5D&cauthor=true&cauthor_uid=21037253), [Digby JE](http://www.ncbi.nlm.nih.gov/pubmed?term=Digby%20JE%5BAuthor%5D&cauthor=true&cauthor_uid=21037253), [Ghimire G](http://www.ncbi.nlm.nih.gov/pubmed?term=Ghimire%20G%5BAuthor%5D&cauthor=true&cauthor_uid=21037253), *et al*. Eur Heart J. 2011;32:856-66.

113. Eshtehardi P, Windecker S, Cook S, *et al*. Am Heart J 2010; 159: 891-8.

114. [Breet NJ](http://www.ncbi.nlm.nih.gov/pubmed?term=Breet%20NJ%5BAuthor%5D&cauthor=true&cauthor_uid=20179285), [van Werkum JW](http://www.ncbi.nlm.nih.gov/pubmed?term=van%20Werkum%20JW%5BAuthor%5D&cauthor=true&cauthor_uid=20179285), [Bouman HJ](http://www.ncbi.nlm.nih.gov/pubmed?term=Bouman%20HJ%5BAuthor%5D&cauthor=true&cauthor_uid=20179285), et al. [JAMA](http://www.ncbi.nlm.nih.gov/pubmed/20179285) 2010;303:754-62.

115. [Libby P](http://www.ncbi.nlm.nih.gov/pubmed?term=Libby%20P%5BAuthor%5D&cauthor=true&cauthor_uid=19942084), [Ridker PM](http://www.ncbi.nlm.nih.gov/pubmed?term=Ridker%20PM%5BAuthor%5D&cauthor=true&cauthor_uid=19942084), [Hansson GK](http://www.ncbi.nlm.nih.gov/pubmed?term=Hansson%20GK%5BAuthor%5D&cauthor=true&cauthor_uid=19942084). J Am Coll Cardiol. 2009;54:2129-38.

116. [Hansson GK](http://www.ncbi.nlm.nih.gov/pubmed?term=Hansson%20GK%5BAuthor%5D&cauthor=true&cauthor_uid=21321594), [Hermansson A](http://www.ncbi.nlm.nih.gov/pubmed?term=Hermansson%20A%5BAuthor%5D&cauthor=true&cauthor_uid=21321594). Nat Immunol. 2011;12:204-12.

117. [Libby P](http://www.ncbi.nlm.nih.gov/pubmed?term=Libby%20P%5BAuthor%5D&cauthor=true&cauthor_uid=21593864), [Ridker PM](http://www.ncbi.nlm.nih.gov/pubmed?term=Ridker%20PM%5BAuthor%5D&cauthor=true&cauthor_uid=21593864), [Hansson GK](http://www.ncbi.nlm.nih.gov/pubmed?term=Hansson%20GK%5BAuthor%5D&cauthor=true&cauthor_uid=21593864). Nature. 2011;473:317-25.

118. Goodchild TT, Robinson KA, *et al*. JACC Cardiovasc Interv. 2009;2:1005-16.

119. [Hofmann U](http://www.ncbi.nlm.nih.gov/pubmed?term=Hofmann%20U%5BAuthor%5D&cauthor=true&cauthor_uid=22388323), [Beyersdorf N](http://www.ncbi.nlm.nih.gov/pubmed?term=Beyersdorf%20N%5BAuthor%5D&cauthor=true&cauthor_uid=22388323), [Weirather J](http://www.ncbi.nlm.nih.gov/pubmed?term=Weirather%20J%5BAuthor%5D&cauthor=true&cauthor_uid=22388323), *et al*. Circulation. 2012;125:1652-63.

120. [Campbell KA](http://www.ncbi.nlm.nih.gov/pubmed?term=Campbell%20KA%5BAuthor%5D&cauthor=true&cauthor_uid=22427326), [Lipinski MJ](http://www.ncbi.nlm.nih.gov/pubmed?term=Lipinski%20MJ%5BAuthor%5D&cauthor=true&cauthor_uid=22427326), [Doran AC](http://www.ncbi.nlm.nih.gov/pubmed?term=Doran%20AC%5BAuthor%5D&cauthor=true&cauthor_uid=22427326), *et al*. Circ Res. 2012;110:889-900.

121. [Gerszten RE](http://www.ncbi.nlm.nih.gov/pubmed?term=Gerszten%20RE%5BAuthor%5D&cauthor=true&cauthor_uid=22551134), [Tager AM](http://www.ncbi.nlm.nih.gov/pubmed?term=Tager%20AM%5BAuthor%5D&cauthor=true&cauthor_uid=22551134). N Engl J Med. 2012;366:1734-6.

122. Bernal-Mizrachi L, Jy W, Jimenez JJ, [Pastor J](http://www.ncbi.nlm.nih.gov/pubmed?term=%22Pastor%20J%22%5BAuthor%5D), *et. al*. Am Heart J. 2003;145:962-70.

123. [van Gils JM](http://www.ncbi.nlm.nih.gov/pubmed?term=van%20Gils%20JM%5BAuthor%5D&cauthor=true&cauthor_uid=22231519), [Derby MC](http://www.ncbi.nlm.nih.gov/pubmed?term=Derby%20MC%5BAuthor%5D&cauthor=true&cauthor_uid=22231519), [Fernandes LR](http://www.ncbi.nlm.nih.gov/pubmed?term=Fernandes%20LR%5BAuthor%5D&cauthor=true&cauthor_uid=22231519), *et al.* Nat Immunol. 2012;13:136-43.

124. [Kyaw T](http://www.ncbi.nlm.nih.gov/pubmed?term=Kyaw%20T%5BAuthor%5D&cauthor=true&cauthor_uid=21868694), [Tay C](http://www.ncbi.nlm.nih.gov/pubmed?term=Tay%20C%5BAuthor%5D&cauthor=true&cauthor_uid=21868694), [Krishnamurthi S](http://www.ncbi.nlm.nih.gov/pubmed?term=Krishnamurthi%20S%5BAuthor%5D&cauthor=true&cauthor_uid=21868694), *et al*. Circ Res. 2011;109:830-40.

125. [Kyaw T](http://www.ncbi.nlm.nih.gov/pubmed?term=Kyaw%20T%5BAuthor%5D&cauthor=true&cauthor_uid=21881498), [Tipping P](http://www.ncbi.nlm.nih.gov/pubmed?term=Tipping%20P%5BAuthor%5D&cauthor=true&cauthor_uid=21881498), [Toh BH](http://www.ncbi.nlm.nih.gov/pubmed?term=Toh%20BH%5BAuthor%5D&cauthor=true&cauthor_uid=21881498), [Bobik A](http://www.ncbi.nlm.nih.gov/pubmed?term=Bobik%20A%5BAuthor%5D&cauthor=true&cauthor_uid=21881498). Curr Opin Lipidol. 2011;22:373-9.

126. [Kyaw](http://www.ncbi.nlm.nih.gov/sites/entrez?cmd=search&db=PubMed&term=%20Kyaw%2BT%5Bauth%5D) T, [Tay](http://www.ncbi.nlm.nih.gov/sites/entrez?cmd=search&db=PubMed&term=%20Tay%2BC%5Bauth%5D) C, [Hosseini](http://www.ncbi.nlm.nih.gov/sites/entrez?cmd=search&db=PubMed&term=%20Hosseini%2BH%5Bauth%5D) H, *et al*. PLoS One. 2012;7:e29371.

127. Instituto Nacional de Fluidos Complexos – INCT-FCx. <http://fluidos.usp.br/>

128. [Monteiro AM](http://www.ncbi.nlm.nih.gov/pubmed?term=Monteiro%20AM%5BAuthor%5D&cauthor=true&cauthor_uid=19254121), [Jardini MA](http://www.ncbi.nlm.nih.gov/pubmed?term=Jardini%20MA%5BAuthor%5D&cauthor=true&cauthor_uid=19254121), [Alves S](http://www.ncbi.nlm.nih.gov/pubmed?term=Alves%20S%5BAuthor%5D&cauthor=true&cauthor_uid=19254121), *et al*. J Periodontol. 2009;80:378-88.

129. Abonnenc M, Stegemann C, Mayr M. Expert Rev Proteomics 2010;7:811–13.

130. Lewis GD, Asnani A, Gerszten RE. J Am Coll Cardiol. 2008;52:117-23.

131. Cavill R, Keun HC, Holmes E, *et al.* Bioinformatics 2008, 25:112-8.

132. Watson AD. J Lipid Res 2006, 47:2101-2111.

133. Lewis GD, Wei R, Liu E et al. J Clin Invest 2008;118: 3503–12.

134. [Wu DJ](http://www.ncbi.nlm.nih.gov/pubmed?term=Wu%20DJ%5BAuthor%5D&cauthor=true&cauthor_uid=22040517), [Zhu BJ](http://www.ncbi.nlm.nih.gov/pubmed?term=Zhu%20BJ%5BAuthor%5D&cauthor=true&cauthor_uid=22040517), [Wang XD](http://www.ncbi.nlm.nih.gov/pubmed?term=Wang%20XD%5BAuthor%5D&cauthor=true&cauthor_uid=22040517). J Clin Bioinform 2011;1:30.

135. Martin JC, Canlet C, Delplanque B. Atherosclerosis 2009, 206:127-33.

136. Zhang F, Jia Z, Gao P. Talanta 2009, 79:836-844.

137. Mayr M, Chung YL, Mayr U. ATVB 2005, 25:2135-42.

138. Li N, Liu JY, Timofeyev V, *et al*. J Mol Cell Cardiol.2009;47:835-45.

139. Liu JY, Yang J, Inceoglu B, *et al*. Biochem Pharmacol. 2010;79:880-7.

140. Bligh EG, Dyer WJ. Can J Biochem Physiol. 1959;37:911-7.

141. Miyazaki H, Ishibashi M, Takayama H, *et al*. J Chromatogr 1984;289:249-58.

142. Ferreira CR, Saraiva SA, Catharino RR, *et al.* J Lipid Res. 2010;51:1218-27.

143. Saric J, Want EJ, Duthaler U, *et al.* Anal. Chem. 2012, 84: 6963−6972.

144. Trygg J, Holmes E, Lundstedt T. J. Proteome Res. 2007, 6: 469-479.

145. [Caricilli AM](http://www.ncbi.nlm.nih.gov/pubmed?term=Caricilli%20AM%5BAuthor%5D&cauthor=true&cauthor_uid=22162948), [Picardi PK](http://www.ncbi.nlm.nih.gov/pubmed?term=Picardi%20PK%5BAuthor%5D&cauthor=true&cauthor_uid=22162948), [de Abreu LL](http://www.ncbi.nlm.nih.gov/pubmed?term=de%20Abreu%20LL%5BAuthor%5D&cauthor=true&cauthor_uid=22162948), *et al*. PLoS Biol. 2011;9:e1001212.

146. [Unwin N](http://www.ncbi.nlm.nih.gov/pubmed?term=Unwin%20N%5BAuthor%5D&cauthor=true&cauthor_uid=12207806), [Shaw J](http://www.ncbi.nlm.nih.gov/pubmed?term=Shaw%20J%5BAuthor%5D&cauthor=true&cauthor_uid=12207806), [Zimmet P](http://www.ncbi.nlm.nih.gov/pubmed?term=Zimmet%20P%5BAuthor%5D&cauthor=true&cauthor_uid=12207806), [Alberti KG](http://www.ncbi.nlm.nih.gov/pubmed?term=Alberti%20KG%5BAuthor%5D&cauthor=true&cauthor_uid=12207806). Diabet Med. 2002;19:708-23.

147. [Lakka HM](http://www.ncbi.nlm.nih.gov/pubmed?term=Lakka%20HM%5BAuthor%5D&cauthor=true&cauthor_uid=12460094), [Laaksonen DE](http://www.ncbi.nlm.nih.gov/pubmed?term=Laaksonen%20DE%5BAuthor%5D&cauthor=true&cauthor_uid=12460094), [Lakka TA](http://www.ncbi.nlm.nih.gov/pubmed?term=Lakka%20TA%5BAuthor%5D&cauthor=true&cauthor_uid=12460094), *et al*. JAMA. 2002;288:2709-16.

148. [Wang J](http://www.ncbi.nlm.nih.gov/pubmed?term=Wang%20J%5BAuthor%5D&cauthor=true&cauthor_uid=17303589), [Ruotsalainen S](http://www.ncbi.nlm.nih.gov/pubmed?term=Ruotsalainen%20S%5BAuthor%5D&cauthor=true&cauthor_uid=17303589), [Moilanen L](http://www.ncbi.nlm.nih.gov/pubmed?term=Moilanen%20L%5BAuthor%5D&cauthor=true&cauthor_uid=17303589), *et al*. Eur Heart J. 2007;28:857-64.

149. [Malik S](http://www.ncbi.nlm.nih.gov/pubmed?term=Malik%20S%5BAuthor%5D&cauthor=true&cauthor_uid=15326067), [Wong ND](http://www.ncbi.nlm.nih.gov/pubmed?term=Wong%20ND%5BAuthor%5D&cauthor=true&cauthor_uid=15326067), [Franklin SS](http://www.ncbi.nlm.nih.gov/pubmed?term=Franklin%20SS%5BAuthor%5D&cauthor=true&cauthor_uid=15326067), *et al.* Circulation. 2004;110:1245-50.

150. Vamos EP, Millett C, Parsons C, *et al*. Diabetes Care. 2012;35:265-72.

151. Nicolau JC, Serrano CV Jr, Giraldez RR, *et al*. Diabetes Care. 2012;35:150-2.

152. Cani PD, Bibiloni R, Knauf C, *et al*. Diabetes; 2008, 57: 1470-81.

153. Creely SJ, McTernan PG, *et al*. Am J Physiol Endocrinol Metab. 2007, 292: E740-47.

154. Tsukumo DML, Carvalho-Filho MA, *et al*. Diabetes. 2007, 56: 1986-1998.

155. Hattori M, Taylor TD. DNA Research. 2009;16:1-12.

156. Haiat PD. Obesidade e intestino. In: Naves A. Nutrição Clínica Funcional – Obesidade. VP Editora. São Paulo. 2009.

157. Kort S, Keszthelyi D, Masclee AAM. Obesity reviews. 2011, 12: 449-58.

158. [American Diabetes Association](http://www.ncbi.nlm.nih.gov/pubmed?term=American%20Diabetes%20Association%5BCorporate%20Author%5D&cauthor=true&cauthor_uid=22187472). Diabetes Care. 2012;35:S64-71.
