# Supplementary material for: Changes in lipoproteins associated with lipid-lowering and antiplatelet strategies in patients with acute myocardial infarction
Source: PLoS One. 2022 Aug 30;17(8):e0273292. doi: 10.1371/journal.pone.0273292 (PMC9426937; doi:10.1371/journal.pone.0273292)
Supplement: S2 File — (DOCX) [file pone.0273292.s004.docx]

**Thematic project**

**ROLE OF INNATE AND ADAPTIVE IMMUNITY IN ISCHEMIC HEART DISEASE AFTER ACUTE MYOCARDIAL INFARCTION**

**Principal investigator:**

Francisco Antonio Helfenstein Fonseca

Lipids, Atherosclerosis and Vascular Biology Laboratory

Cardiology Division

Escola Paulista de Medicina

Universidade Federal de São Paulo

2012

**ROLE OF INNATE AND ADAPTIVE IMMUNITY iN THE ISCHEMIC HEART DISEASE AFTER ACUTE MYOCARDIAL INFARCTION**

**1. ABSTRACT**

For hundreds of thousands of years, part of our genomic heritage was primarily developed to combat infectious agents. However, our immune system modulating the progression of atherosclerosis was a challenge much more recently recognized. The fascinating and differential role of lymphocyte subtypes in the development of coronary artery disease may be a new strategic target for understanding and therapy of acute myocardial infarction. The muscle loss in the first hours of coronary occlusion determines important prognostic role and the coronary reperfusion either with thrombolytic agents or percutaneous intervention are currently the strategies of choice for these patients. However, surrounding the necrotic core, an ischemic area is formed, that progresses to extension of the infarction or recovers, mainly during the next three weeks after the ischemic insult. The determinants of cell viability are unknown, postulating that they arise from factors not only related to microcirculation or energy expenditure, but also to inflammatory and immune responses. Furthermore, the intense mobilization of progenitor cells secondary to myocardial infarction triggers large lymphocyte mobilization that colonizes plaques in development, contributing to recurrence of ischemic events. This project aims to understand the immune and metabolic mechanisms involved in the recovery of the ischemic myocardium and coronary disease progression. Specifically, the innate and adaptive immunity will be studied, with emphasis on lymphocytes subtypes involved in the coronary heart disease of patients with acute myocardial infarction. In addition, the project will evaluate biomarkers identified by metabolomics studies, as well as the corresponding signaling pathways. Therapeutic pharmacological strategies and changes on intestine microbiota will be evaluated since the acute phase of myocardial infarction up to six months, based on the MRI studies with late enhancement performed in the acute and late follow-up.

**2. PROBLEM STATEMENT**

This Project will allow further research of the Lipids, Atherosclerosis, and Vascular Biology Laboratory of the Discipline of Cardiology at *Escola Paulista de Medicina* - *Universidade Federal de São Paulo*, in the understanding of mechanisms related to tissue repair after acute myocardial infarction.

The Lipids, Atherosclerosis, and Vascular Biology Laboratory was created in 1998 and it is part of the Cardiology Discipline at *Escola Paulista de Medicina*. The sector develops experimental studies and clinical trials since its foundation, integrating physicians, nutritionists, biomedicals, biologists, veterinarians, physicists, and chemists, allowing a multidisciplinary approach in their researches. With these characteristics, the group established solid interfaces, employing gradually more sophisticated tools for the understanding of the atherosclerosis development and therapeutic mechanisms for its control.

Researches foccused on the endothelial repair after injury by balloon-catheter were the initial experiments developed in the Biophysics Department of the Escola Paulista de Medicina and subsequently in suine model at the Mount Sinai School of Medicine in NYC. Several studies were carried out involving vasoreactivity in vivo and in vitro.^1-4^

Clinical studies involving vasoreactiity were further developed ^5,6^ and we started to explore the contribution of circulating endothelial progenitor cells and several microparticles ^7,8^. In parallel, we started to examine morphological and therapeutic aspects of atherosclerosis ^9-12^.

In recent years, we have incorporated the view of atherosclerosis as an inflammatory and immune disease ^13-23^. In this context, through the participation in two institutes (Millenium Institute of complex fluids and National Institute of Science and Technology of Complex Fluids) we started to study more deeply the immune responses triggered by oxidized lipoproteins and the balance of cholesterol synthesis and absorption, depending of the strategy employed (pharmacological or non-pharmacological) ^24-28^.

Clinical trials, case-control studies, studies involving therapeutical approaches, as well long-term observational studies were developed, and in many of them we were able to examine genetic variants and molecular biology procedures, such as gene expression^29-58^.

More recently, we started to study pharmacokinetic interactions among drugs commoly used in the acute phase of myocardial infarction, and we found interesting interactions between statins and antiplatelet agents^8,59^, exploring some pleiotropic effects in the first hours of therapy, such as the remarkable improvement of endothelial function, or revealing increased circulating statin and thienopyridine concentrations inversely related to the number of circulating platelet microparticles.

Currently, atherosclerosis has been understood as a chronic inflammatory and immune disease, involving the humoral and cellular compartments of the innate and adaptive immunity. These responses have been demonstrated by the autoantibodies and immune complex found in atherosclerotic lesions^60^. The innate responses are mainly due to of monocytes/macrophages and dendritic cells, in initial or advanced plaques ^61^. These cells are usually proatherogenic, although some have anti-atherogenic role^62^. Interestingly, B1 cells found in many tissues such as spleen, intestine, peritoneum, and pleural cavity can be differentiate in B1a, B1b, Breg and B10 ^63^. The dendritic cells are related to differentiation of T cells into specific subtypes (Th1, Th2, Treg) which may determine pro-aterogenic (Th1) or protective (Treg) responses^64^.

An important aspect was the report that the adventitia may contain a large population of B cells, forming the so-called tertiary lymphoid organ^65^. While B1 cells are considered ateroprotectives, mainly due to the production of IgM anti oxidized LDL, B2 cells are considered pro-atherogenic due to the release of IgG related to immune complex deposition in the intima vascular layer^66^.

More recently, the macrophage eflux from the intima layer was reported, involving a signalling related to CCL19, CCL21 and CCR7 receptor, possibly via *vasa* *vasorum*^67,68^. It is clear that the presence of B cells in this vascular region is implicated in physiological defense, associated to the neovascularization and that the macrophage eflux is a dinamic process, mediated by immune cells in the adventitia.

It has been suggested that increased cholesterol diminishes the macrophage eflux, favoring their residence in the vascular intima, and plaque complications, thus potential benefits can be provided by statins^69^. Finally, experimental therapy using B cells was effective to repair ischemic tissue, improving ventricular remodeling^70^.

There is great recurrence of cardiovascular events after myocardial infarction, and in this sense, the mobilization of lymphocyte cells in response to the myocardial lesion determines hgher colonization in atherosclerotic plaques established in other vascular sites, explaining the high rates of recorrent cardiovascular events, particularly in the first year after an acute coronary syndrome ^71^.

Our main purpose in this thematic project is to characterize subtypes of B lymphocytes and to determine their role in ventricular remodeling at short and long term after acute myocardial infarction. Besides that, the study will explore early and late effects in the microcirculation (cardiac magnetic resonance imaging with late enhancement) and ventricular function. Recently, we reported differences in the total IgG against oxLDL by the comparison of rosuvastatin with simvastatin/ezetimibe, despite comparable decrease in the LDL-C ^72^. In a pilot study, we quantified B1 (CD70-CD19+CD20+CD27+) and B2 (CD23+CD19+) lymphocytes, in addition of T CD4 and CD8 lymphocytes, with samples collected in the first hours of acute myocardial infarction, that were compared with healthy controls. We found increased percentage of B2 cells and trends for reduced B1 and T CD8+ cells, showing interesting data in atherosclerosis and plaque instability^73,74^ .

Among these estrategies, we will examine the synergism between statins and antiplatelet drugs, changes in the microbiota and their effects in lymphocyte differentiation, the evolution in the ischemic disease, biochemical parameters and metabolomics. The substudies described below show the basis for the proposed therapies, their originality and clinical relevance.

***SUBPROJECT 1***

**EfFECTS OF THE INTERACTION BETWEEN STATINS AND ANTIPLATELETS IN THE EVOLUTION OF ACUTE MYOCARDIAL INFARCTION**

**Summary**

The coronary occlusion determines the loss of myocardium mass in the first hours of acute myocardial infarction. However, around the necrotic tissue, the ischemic myocardium may recover or progress to the infarcted mass in the following 3-4 weeks with prognostic implications at long term. Functional endothelial recovery seems fundamental for the improvement of microcirculation and ventricular remodeling. This substudy will examine possible benefits from the interactions between statins and antiplatelets regarding antithrombotic and vasodilatation properties. Patients with ST segment elevation myocardial infarction (STEMI), treated in the first 6-hours with pharmacological thrombolysis and referred to the hospital for coronary angiogram, will be randomized by 2x2 factorial design for treatment with rosuvastatin 20 mg qd or simvastatin 40 mg plus ezetimibe 10 mg qd, as well as for ticagrelor 90 mg bid or clopidogrel 75 mg qd, and will also receive the conventional therapy for patients with STEMI. The treatment randomly allocated will be kept for six months and during this period cMRI with late enhancement will be performed in the first 3-5 days, and repeated at 4 weeks and six months to quantify myocardial necrosis, ischemic tissue and to evaluate the ventricular function.

**1. Proposal rationale**

The first study with statin, involving coronary patients to analyse cardiovascular clinical events, enrolled subjects with NSTEMI, and atorvastatin was initiated only between 24 and 96 hours after MI^75^. Further, the PROVE-IT study^76^, compared atorvastatin with pravastatin, but the lipid-lowering agents were started after the 10^th^ day, thus examining the intervention in a later period. On the other hand, registries in US^77,78^ have shown decrease in-hospital mortality related to the use of statins started in the first 24 hours of AMI. Furthermore, decrease in heart failure was associated with the use of statins started in the first 24 hous, in patients treated by pharmacological thrombolysis as well as those treated by primary angioplasty^79^.

In the ARMYDA-RECAPTURE study^80^, high doses of atorvastatin prescribed few hours before coronary intervention were associated with lower levels of markers of myocardial injury (troponin and CKMB) and better survival free of events in the first month, suggesting that pleiotropic effects and not lipid changes were the main determinants for these benefits. More recently, study with rosuvastatin 40 mg pre percutaneous coronary intervention showed better evolution for the main cardiovascular events, following 445 patients up to 12 months (9,8% vs. 20,5%, p=0,002)^81^.

Simvastatin is frequently prescribed in hospitalized subjects with AMI. However, as a pro-drug, its pleiotropic effects can be reduced by the concomitant prescription of thienopyridines and other drugs of potential pharmacokinetic interactions, delaying the formation of actives metabolites.

Clopidogrel is a prodrug that need extent metabolization by the cytochrome P450 until the formation of the thiol active metabolite. Therefore, drugs that compete for the isoenzymes CYP 1A2, 2C9, 2C19 e 3A4, have potential for pharmacokinetic interactions with clopidogrel. Recently, we examined the serum levels of concomitant prescription of clopidogrel 75 qd and atorvastatin 80 qd, in stable patients with coronary disease and an interaction between these drugs was observed showing increased concentrations of atorvastatin and decreased concentrations of clopidogrel, without lack of antiplatelet aggregation with clopidogrel or in the lipid-lowering effects of atorvastatin. However, there was an inverse relationship between the number of circulating platelet microparticles and serum concentrations of clopidogrel (AUC e CMax)^8^. In other study, we reported increased concentrations of rosuvastatin after the addition of clopidogrel (300 mg) but much lower effects with 75 mg. ^59^ Improvement in the endothelial function was observed after 24 hours of statin treatment and there was synergic effect in the antiplatelet analysis and remarkable lipid-lowering effect. However, partial loss of these benefits were seen one week of statin withdrawal, despite the continuous use of the thienopyridine, due to significant increase in the circulating platelet microparticles^59^. Taken together, these data suggest that the use of rosuvastatin (20 mg) may be associated with early benefits in subjects with acute coronary syndromes through the potential synergism in antithrombotic effects and improvement in the microcirculation.

Regarding ticagrelor, this antiplatelet agente is the first to reversibly block P2Y12 receptor, with faster platelet inhibition in comparison with clopidogrel^82^. Furthermore, unexpected benefits in mortality, observed in the PLATO study^83^, but not with other antiplatelet agent with high antiplatelet effect in comparison with clopidogrel, the prasugrel in the TRITON-TIMI 38 study^84^, suggested that ticagrelor may have other benefits beyond its antiplatelet effects^85-87^. In fact, recent publication showed that ticagrelor, but not clopidogrel, avoid the muscle contraction induced by ADP^88^. Thus, it is possible that increase in adenosine concentrations mediates the mechanism of ticagrelor benefits, improving microcirculation and allowing recovery of ventricular function. In addition, the reversibility of the interaction of ticagrelor with the P2Y12 receptor may avoid bleeding contributing to reduced cardiovascular mortality.

The myocardial stunning is related to prolonged decrease in myocardial contractility due to ischemia, despite reperfusion^89^, while the myocardial hibernation is commonly employed to report a more persistent contractility dysfunction due to coronary artery disease^90^. The mechanisms involved with the last are less understood and not always the perfusion is reduced in the areas of myocardial hybernation. Recent studies have shown that cMRI is a very promising imaging method to detect myocardial viability^91^. The method is automatized, has great reproducibility, and even with the literature still restrict, small studies have shown prognostic value with respect to the infarcted mass after coronary interventions^92,93^. The functional recovery of ischemic myocardial and no-reflow studies will complement the cMRI analysis of special interest in these patients.

Taken together, with this subproject, we will evaluate if the early and optimized therapy with rosuvastatin and ticagrelor determines immediate benefits in the coronary microcirculation with favorable ventricular remodeling in the first weeks after STEMI. Simvastatin is commonly used in subjects with AMI, but due to be a prodrug, it can be less effective in the presence of other thienopyridines due to the delay for its liver metabolization.

**2. Objectives**

**2.1. General objectives**

- - 1. To compare the four therapeutic strategies in the ventricular function and infarcted mass by cMRI early (3-5 days) and in two late times (after 4 wk and 6 mo).

**2.2. Secondary objectives**

- - 1. To evaluate the safety and tolerability of the therapeutic strategies, through the biochemical analyses (changes in the lipid profile, muscle and liver enzymes and cardiac biomarkers).
    2. To evaluate major and minor bleedings^80^.

1. **Methods**

The study will include patients with STEMI treated by pharmacological thrombolysis in the first 6 hours of onset of symptoms. Patients of both sexes with age of 75 or less will be included. Exclusion criteria includes comorbidities that may affect the use or safety of the study drugs, such as active liver disease, recente bleedings, neoplasias, cardiogenic shock, known intolerance of the study drugs, or personal history of alcoholism or other condition that compromise an appropriate evaluation of the treatments (drug addition, infectious disease, rheumatological diseases, AIDS, LES, rheumatic arthritis).

The study design is PROBE (prospective, randomized, open label trial with blinded endpoints). Sample size was estimated based on the previous findings by cMRI pre and after coronary reperfusion, showing that the method is able to identify áreas of myocardial recovery^94^. Thus, it was estimated that infarcted patients have approximately 20% of ischemic myocardium around the necrotic area that contributes to hypocontractility, corresponding to viable myocardium. Sample size was estimated on basis of the expected diferences between groups (10-20% in the infarcted mass with 90% of power and alpha risk of 5%, considering 30% of standard deviation). Thus, the sample size was 223 patients in the worse scenario (Minn M. Soe and Kevin M. Sullivan, Emory University). As we did not have previous data of these pharmacological strategies and considering the 2x2 factorial design, we decide to include 300 patients (75 patients for each study arm). The cMRI data will be analyzed by two experient specialists in these modalities of imaging. Intra and inter variability among these investigators will be performed at each 1/3 of data obtained. The study will be registered as clinical trial before starting and will comply with the international standards for good clinical practice and data harmonization (GCP/ICH).

**Cardiac Magnetic Resonance Imaging with late enhancement**

The cMRI will be performed to identify and to quantify the area of necrosis and ischemia. The exams will be performed in equipments 3.0 T scanners and protocol included steady-state free precession imaging (SSFP) for anatomical evaluation, retrospective cine imaging in long and short axis views and late contrast-enhanced imaging using gadolinium for evaluation of myocardium scar/fibrosis. Cine images will be obtained in the two-chamber, four-chamber, left ventricular outflow tract, and short-axis views with the first slice positioned at the mitral valve and last covering the apex, resulting in 10-12 cine breath-hold short-axis images to cover the entire ventricle. Infarcted mass will be quantified using myocardial delayed enhancement technique, after the injection of gadolinium-based contrast agent. Contrast-enhanced images will be acquired in the same views as those used for cine MRI, with the use of a segmented inversion-recovery sequence. The necrotic tissue mass will be estimated in grams and as the percentage of the necrotic tissue of the left ventricular mass using the 17 myocardial regions according the American Heart Association classification^95^. Segmentar contractility will be quantified , as well as diastolic and systolic volume, and ventricular ejection fraction^96-104^. Myocardial perfusion will be analyzed initially without stress, and with dipiridamol at 4 wk and 6 mo^104^. Each patient study will be reviewed by two independent blinded readers using dedicated software.

***SUBPROJECT 2***

**ROLE OF ENDOTHELIAL PROGENITOR CELLS, ENDOTHELIAL AND PLATELET MICROPARTICLES IN THE EVOLUTION OF ACUTE MYOCARDIAL INFARCTION**

**Summary**

Classic risk factors for coronary disease such as diabetes, hypertension, smoking and hypercholesterolemia, reduce the circulating endothelial progenitor cells (EPC) and are related to higher rates of endothelial cells apoptosis and thrombotic risk. Statins can increase the mobilization os EPC from bone marrow, particularly after percutaneous coronary interventions. Prospective studies suggest that the EPC rates add prognostic information, but less is known related of the role of microparticles. Considering that coronary occlusion is usually associated with dysfunctional endothelium, the faster anatomical and functional recovery of these cells may be related to the improvement of microcirculation and recovery of the ischemic myocardium. Recently, study from our group showed an interaction between statins and clopidogrel not only in the serum levels of these drugs, but also in the circulating rates of endothelial and platelet microparticles. Thus, with this subproject, we will explore possible benefits of the interaction between clopidogrel, ticagrelor, rosuvastatin and sinvastatina/ezetimibe, in the decrease of the ischemic myocardium around the necrotic tissue in the first weeks after STEMI. Our hypothesis is that the pharmacological strategies with statins and antiplatelet be associated with mobilization of EPC and affect the circulating microparticles rates. Studies involving the flow-mediated dilation (FMD) and by cMRI will explore these hypothesis. Using specific CDs, the cellular elements described will be quantified by flow-cytometry, with methodology already established in our laboratory. The FMD will follow the recommendations of the European Society of Cardiology and the patients will be those included in the subproject 1, using the 2x2 factorial design.

1. **Proposal rationale**

The anatomic and functional endothelium plays important role in the cardiovascular disease. With advancing age, a profound change in the endothelial phenotype has been reported, acquiring pro-inflammatory, pro-oxidant, and pro-proliferative characteristics^105^. Classic risk factors may impair the adequate replacement of the senescente or apoptotic endothelium, favoring higher risk of thrombotic occlusions^106^. As the anatomic absence of the endothelium is not identified by conventional angiography, this condition seems subestimated. Oxidized LDL, inflammatory cytokines and lower hemodynamic shear stress, can stimulate endothelial apoptosis, and should be considered physiological responses, but when excessive, are related to higher risk for cardiovascular events^107^. Recent publication revealed that the infusion of CD34+ cells (one characteristic of EPC) was associated with both improvement of myocardial perfusion and cardiac performance six months after AMI^108^. Another study showed improvement in the cardiac function by cMRI in subjects with higher circulating rates of CD34+ cells^109^.

Recent study from our group found that withdrawal of statins for one week is followed by significant increase in circulating platelet microparticles, despite the continued use of clopidogrel, in patients with coronary heart disease^8^. Furthermore, substantial improvement in the endothelial function was reported 24 hours after the first dose of rosuvastatin, also in patients with coronary heart disease^59^. Among other subgroups of patients, such as subjects HIV+ naive of antiretroviral therapy we reported an imbalance between EPC and endothelial microparticles associated to endothelial dysfunction (FMD), suggesting that the viral load may be related to imbalance in the turnover of endothelial cells, revealing a new mechanism to explain the high rates of cardiovascular disease in these patients^7^.

1. **Objectives**
   1. **General objetives**
      1. To quantify EPC, endothelial (EM) and platelet (PM) microparticles at baseline, and after 30 and 180 days after STEMI.
      2. To evaluate possible diferences in EPC, EM, and PM according to the pharmacological strategies (four study arms).
   2. **Secondary objectives**
      1. To examine FMD during hospitalization and after 30 and 180 days after STEMI.
      2. To evaluate characteristics of the coronary angiogram such as coronary anatomy, reperfusion, myocardial blush, no-reflow and other characteristics of coronary angiography and possible associations with FMD, CEP and microparticles.
      3. To analyze the cMRI parameters in subjects with no-reflow (estimated to be found in 10% of these subjects) compared with those without no-reflow (case-control patients 1:3).
2. **Methods**

EPC, and microparticles (MP) will be quantified by flow-cytometry, using specific CDs in fresh samples obtained at the mentioned times. These analyses were standardized in our lab and is breafly describe below^7,8,59^.

*Endothelial progenitor cells*

Approximately 15 mL of blood will be collected using EDTA tubes. Ficoll-Hypaque will be added (Ficoll Paque Plus, GE Healthcare Bio-Sciences AB, Uppsala, Sweden) and centrifuged at 2000 rpm, 20-22ºC for 20 minutes, to obtain mononuclear cells separation by concentration gradient. Then, the obtained cells will be washed in isotonic solution (PBS) and the cellular viability analyzed in hemocytometer, after staining of 10 uL of these cells with 90 uL of trypan blue 60% (Sigma-Aldrich, MO, USA) for 5 minutes. Next, the samples are centrifuged and immunomarked for 15 minutes at room temperature using the following antibodies: CD34 conjugated with fluorescein isotiocianate – FITC (BD Biosciences, Franklin Lakes, NJ, USA), KDR conjugated with phycoerythrin - PE (R&D Systems, Minneapolis, USA) e CD133 conjugated with alophycocyanin – APC (Miltenyi Biotec, Auburn, CA, USA). Controls will be analyzed as cells marked with the isotypes IgG1 FITC (BD, Biosciences, Franklin Lakes, USA), IgG1 PE (R&D Systems, Minneapolis, MN) and IgG1-APC (Miltenyi Biotec, Auburn, USA). The flow-cytometer analyses are performed in the FACSCalibur cytometer (BD Biosciences, San Jose, USA) using software Cell Quest Pro, at baseline and after pharmacological strategies in the mentioned timepoints. The identification and quantification of EPC is made in the lymphocytes area, due the similarities in morphology, with the values expressed as percentages (%) of EPC.

*Endothelial and platelet microparticles*

Blood samples collected in tubes with citrate will be centrifuged at 4000 rpm, 20-22ºC for 10 minutes to obtain platelet rich plasma (PRP). The PRP is then centrifugated at 13000 rpm, 20-22ºC for 6 minutes, to obtain poor platelet plasma (PPP). Next, 50 uL of PPP is doubly marked for 20 minutes, at room temperature with anti-CD42 conjugated with FITC and the anti-CD31 conjugated with PE (BD Biosciences, Franklin Lakes, NJ, USA). The CD31 and CD42 are constituints of platelet microparticles. The endothelial microparticles will be quantified using CD51 antibody conjugated with FITC and anti-CD144 conjugated with PE (BD Biosciences, Franklin Lakes, NJ, USA). For controls will be used the isotypes IgG1 FITC and IgG1 PE (BD Biosciences, Franklin Lakes, NJ, USA). These microparticles will be quantified in microliters of PPP which is injected in the flow-cytometer.

TruCOUNT (Becton Dickinson) tubes with known amount of microspheres (beads) will be used to quantify the amount of microparticles per microliter of PPP.

The endothelial function will be estimated by FMD using ultrasonography with high resolution linear transducer using a protocol suggested by the European Society of Cardiology (ESC)^111,112^ using automated analysis by VIVID 7 (GE Healthcare, USA), by two experients ultrasonographer.

***SUBPROJECT 3***

**Study of the immune responses and LDL oxidation in the evolution of acute myocardial infarction**

**Summary**

Atherosclerosis is now considered an inflammatory vascular disease, strongly influenced by the innate and adaptive immunity^113-115^. These studies suggest that some subtytes of lymphocytes may modulate physiological repair responses and other subtypes can aggravate the signalling responses related to thrombotic coronary events. Furthermore, the adaptive immunity seems to influence the ventricular remodeling based in experimental myocardial infarction studies^116,117^. The participation of the *vasa vasorum* in atherosclerosis has been reviewed^118^ and appears to have a notably antiatherosclerotic role, allowing the macrophage efflux from the vascular intima^119^. Hypercholesterolemia seems to decrease the cellular signaling related to this eflux^119^, a condition influenced by statins (direct effect on hypercholesterolemia and indirect pleiotropic effect). In addition, an interaction between B1 lymphocyte with circulating oxidized LDL and related to the B2 lymphocyte with immunecomplex deposition in the intima layer was reported^120-126^. In our institution, the peptide D (synthetized from the apolipoprotein B) has great amphipathic characteristic and it is related to oxidation, and the antibodies against this peptide will be examined.

Previous studies from our group have evidenced that the estimation of imune responses through the IgG antibodies against apolipoprotein B, including those responses to the peptide D are related to the degree of atherosclerosis^29^, to blood pressure levels in hypertensive subjects^28^, to the treatment of arterial hypertension^17^ as well as to the clinical instability in subjects with coronary heart disease^33^. Furthermore, analysis of the degree of lipoprotein oxidation by Laser Z-scan constitutes one of the objectives of the *Instituto Nacional de Ciência e Tecnologia – Fluidos Complexos* (INCT-FCx), of which we are part^127^. Through the diffusibility of the laser beam, quantitative data are obtained to estimate oxidative changes in lipoproteins, which are associated with imune responses to antibodies against oxLDL^128^.

Therefore, in this project we intend to evaluate the imune responses examining subtypes of circulating lymphocytes (mainly B1 and B2), and also the antibodies against oxLDL and the degree of oxidation through the laser beam, seeking better understanding of the role of adaptive immunity related to ventricular remodeling, myocardial necrosis, the severity of coronary disease and also the cardiovascular events during the follow up of these patients with STEMI. For these study, samples from 120 patients included in the subproject 1 will be analyzed and followed for up to a year. Therefore, we will analyze the mentioned parameters at hospitalization, and after 30, 180, and 360 days after AMI. Recently, new and fascinating mechanism was reported to explain the high rates of cardiovascular recorrent events in the first year of these patients, in response to the myocardial necrosis^71^. The AMI determines great mobilization of lymphocytes from the lymphoid tissues, including the spleen, that infiltrates atherosclerotic plaques in development in other vascular sites than the culprit lesion. In other words, the AMI accelerates atherosclerosis and recorrent events are possibly related to acute destabilization of these plaques colonized by these inflammatory lymphocytes. Thus, this subproject also will contribute to better understanding of the role of these cells, by the quantification of lymphocytes subtytes during follow up.

1. **Proposal rationale**

The oxidized LDL constitutes an vascular insult that triggers an immediate response mediated by our innate immune system. On dependency of the cells involved we may have an inflammatory response of the higher or lower intensity. However, our adaptive immunity is much more specific and evolved and also has a relevant role in the modulation of atherosclerosis. Thus, this immune response may determine a reparative healing, involving B lymphocytes and oxidized LDL.

**2. Objectives**

**2.1. General objectives**

2.1.1. To characterize and quantify B lymphocytes by flow-cytometry at hospital admission, and after 30, 180 and 360 days after AMI.

2.1.2. To correlate B lymphocytes with the severity of atherosclerosis, degree of myocardial necrosis, and early and late ventricular remodeling

**2.2. Secondary objectives**

2.2.1. To quantify oxLDL antibodies during hospitalization (3-5 days) and 30 and 180 days after AMI.

2.2.2. To examine oxidative parameters obtained by Laser beam

2.2.3. To verify the association between adaptive immune parameters with cardiovascular events during the follow-up up to 180 days after MI.

**3.Methods**

B1 and B2 lymphocytes will be quantified by flow-cytometry using specific CDs.

*Flow-cytometry for determination of lymphocytes subtypes*

Blood samples (10 mL) will be collected in tubes with heparin. Next, equal volume of phosphate buffer (PBS) will be added and this mixture will be gently placed in Ficoll/hypaque (density 1.077). For each 3 mL of Ficoll will be added 10 mL of the mixture. The sample will be centrifugated at 1300 rpm for 30 minutes. Three phases will be obtained being the mononuclear cells in the interface between the plasma (superior) and polymorphonuclear cells (bottom). The cells will be removed and submitted to three washed steps with PBS and then their viability will be examined by the Neubauer chamber using trypan blue.

*Determination of lymphocyte populations via specific markers by flow-cytometry*

The cells (1 x 10^6^ célls per tube) will be marked by monoclonais antibodies conjugated with fluorochrome. Next, they will be incubated for 1 hour at 4ºC with 1 μL of specific antibodies, defining the following cell populations: anti-CD19 conjugated with PE, anti-CD5 conjugated with PE-Cy7, anti-CD27 conjugated with APC, anti-CD70, anti-CD20 and anti-CD43 for B1 cells; anti-CD19 conjugated with PE and anti-CD23 conjugated with FITC for B2 (CD19^+^ CD23^+^) cells and anti-CD3, anti-CD4 e anti-CD8 for the T lymphocytes. After this period a new washed will be performed, and the cells will be fixed, by adding 100 μL of PBS/BSA 1% adn 400 μL of paraformaldehyde 1%, kept at 4ºC for 30 minutes. After new washed step, the cells will be suspended in 1 mL of PBS and analyzed in flow-cytometer (FACSCANTO –BD or ATTUNE –LIFE Technologies).

The analyses will be made as percentages of cells obtained in the dotplots, accordingly with the cytometer program and then transformed in absolute number based on the white blood cell count. New experiments can be made to characterize new subtypes of lymphocytes.

Antibodies against oxidized LDL as well as for the peptide D will be quantified by ELISA.

*Antibody detection against oxidized LDL (oxLDL)*

To quantify the autoantibodies against oxLDL, will be used the standartized ELISA developed in the prof. Magnus Gidlund laboratory (Fernvik et al., 2004). 96-well plates (Costar, USA) will be sensitized with 50 μL, with 7,5 μg/mL of oxLDL in sodium carbonate buffer 0,1 M, pH 9,4, during 18h, at 4°C. After 4 washed cycles of with 100 µL PBS, the plates will be blocked with gelatin 1.0% solution (Gibco, USA), at room temperature for 24 hours. Next, plates will be washed 4 times with PBS and the wells will be filled (in triplicate) with 50 μL of the samples of different groups, diluted 1:400 in PBS. After 2 hours of incubation, the plates will be washed 4 times with 100 µL of PBS-T and incubated with 50 μL of peroxidase conjugated, for 1 hour, at room temperature. A conjugated immunoglobolin gout IgG anti-human IgG marked with peroxidase (KLP, USA) will be used in a dilution 1:1000. Next, 4 washed cycles with PBS-T, and the analysis will be made after the addition 75 µL de solução de TMB (250 µL de 3,3’5,5’-tetrametilbenzidina 6,5% in DMSO, 12 mL of citrate buffer 0,1M, pH 5,5 e 10 µL de H_2_O_2_ ) in each well. The reaction will stop after the addition of 25 µL of sulfuric acid 2M (Merck, Alemanha). The results will be obtained by espectophotometric at 450 nm using ELISA reader (GENIOS TECAN, Austria). To compensate some imprecisions that might occur in the quantification of intraplaque antibodies by ELISA, we will use in all plates human purified IgG as control (purified human IgG – 10 mg/ml – Pierce Protein Research Products, Thermo Scientific, Rockford). The titers of antibodies will be expressed as index of reactivity (IR) for each sample and calculated as: (Abs of sample – Abs of blank)/( Abs of IgG control - Abs of blank).

The oxidation of LDL will be estimated by the difusibility coefficient to the laser beam after ultracentrifugation of the lipoproteins using a technique developed in the *Instituto de Física* – USP)^125^.

Approximately 120 patients will be included in the study, 30 in each arm, accordingly to the interventions reported in the subproject 1.

***SUBPROJECT 4***

**Study of metabolytes involved in the evolution of the acute myocardial infarction**

**Summary**

Metabolomics and lipidomics are new branches of the science and provide, in conjunct, a well accurated and dynamic portrait of our biological system integrated to bioinformatics. The platforms for liquid chromatography/mass spectrometry (LC-MS/MS) allow nucleotides, aminoacids, organ acids, carbohydrates and lipids, to be analyzed. Thus, this technology allows the identification of analytes related to cardiovascular disease and its evolution, making possible better understanding of the metabolic pathways involved^129-132^. Acute changes related to myocardial infarction have been recently reported^133^, but were restrict to small number of patients included in study of alcohol ablation for treatment of hypertrophic cardiomyopathy. This Project will analyze metabolomic analytes related to the evolution of myocardial infarction. Metabolomics and lipidomics have been proposed as important tools to better comprehension of the inflammatory signaling involved in atherosclerosis^134-138^ and seem adequate to the evaluation of clinical and nutrional therapies proposed for the treatment of this disease. In this Project, the analytes detected since de acute phase of MI and in the long-term evolution will be examined with emphasis for their association with clinical and imaging parameters.

**Proposal rationale**

The main determinants of the functional and anatomic myocardial recovery after acute myocardial infarction are poorly understood^138,139^. The use of metabolomics in the evolution of great number of individuals may minimize some expected bias with small samples of individuals due to comorbidities, age, gender or concomitant therapies. Including selected patients with myocardial infarction, the analysis of several analytes in relation to specific parameters, such as ventricular function, amount of myocardial necrosis, pharmacological strategies and clinical evolution, may identify new biomarkers. The comparison between ticagrelor and clopidogrel will allow better analysis of the role of adenosine in the amount of myocardial fibrosis and ventricular ejection fraction. In addition, some biomarkers of the pleiotropic effects of statins may reveal differences between rosuvastatin and simvastatin.

**1. Objectives:**

**1.1. General objectives:**

1.1.1. To quantify metabolomic analytes at hospitalization and after 30 and 180 days after AMI and to examine their association with myocardial necrosis and ventricular function.

**2. Secondary objectives**

2.1. To verify the correlation between myocardial necrosis and adenosine titers.

2.2. To examine correlation between metabolytes related to small proteins involved in the cell signalling dependent of statins and parameters of cMRI.

**3. Methods**

**3.1.** Metabolomic analytes will be quantified by LC/MS-MS.

*Metabolomics*

Blood samples will be collected at same timepoints of the study design. Samples will be centrifuged to precipitate the protein phase. The supernatant (serum) will be transfered to clean eppendorfs and storaged at -20ºC until metabolomic analysis. Samples for quality control (QC) will be prepared by mixture of 5 μL of each individual sample and will be used to evaluate the instrumental stability during the spectral data acquisition.

All samples will be analyzed by UPLC-MS/MS using high efficient chromatographic system (UPLC Acquity, Waters Ltd., Elstree, U.K.) coupled to a hybrid mass spectrometer of high resolution (MicroMass, Waters MS Technologies Ltd., Manchester, U.K) with analyzer Q-TOF, via electrospray ionization, using columns of reverse phase Waters Acquity UPLC BEH C18 (1,8 μm, 2,1 x 100 mm) and normal phase (hydrophylic interaction) Waters Acquity HILIC BEH (1,7 μm, 2,1 x 100 mm), operated at 50°C and 40°C, respectively. For the column C18: A = formic acid 0,1% in water, B = formic acid 0,1% in methanol. Gradient elution with flow 0,4 mL/min: 0-2 min, 99,9% A:0,10% B; 6 min, 75% A:25% B; 10 min, 20% A:80% B, 12 min, 10% A:90% B, 21-23 min, 0,10% A:99,9% B, 24-26 min: 99,9% A:0,10% B. For the column HILIC: A = 95% acetonitrile: 5% ammonium acetate 200 mmol/L, containing a total of 0.1% formic acid; B = 50% acetonitrile: 50% ammonium acetate 20 mmol/L, having a total of 0,1% formic acid. Gradient elution with flow 1,4 mL/min: 0-1 min, 99% A:1% B; 12 min, 100% B; 12,1-15 min, 99% A:1% B. Strong solvent will be 5% acetonitrila and weak solvent will be 95% acetonitrile, both used in the wash of the automatic injector needle.

Common chromatographic conditions to both inclued temperature of the autosampler (4°C) and volume of injection (5 μL). Starting conditions for the mass spectrometer include: capillary tension of 3200 V (positive ionization) or 2400 V (negative ionization), cone tension of 35 V, desolvatation temperature at 350°C, source temperature at 120°C and gas flowsof 25 L/h in cone and 900 L/h in desolvatation. The Q-TOF will be operated with V optics and data acquisition velocity of de 0,2 s e interscan delay of 0,01 s. An encephaline leucine (*m/z* 556,2771) 200 pg/μL em 50% acetonitrile will be used as lockmass, being directely inserted in the instrument with flow 3 μL/min, via an auxiliary sprayer. Data will be collected in the centroid mode in the range of 50 – 1000 *m/z*, with lockmass scans collected at each 15 s, with a mean of 3 scans to be used to mass correction.

The raw data obtained by UPLC-MS will be analyzed by the software XCMS (version 1.24.1) in the R platform, providing a feature table (aligned and normalized data), showing *m/z*, retention times and intensity peaks, for each sample. Data will ve evaluated by multivariate analysis, including principal componente analysis (PCA), projection to latent structure – discriminant analysis (PLS-DA) and ortogonal (O-PLS-DA)^139-142^ The identity of the metabolytes will be obtained by research using available database: Human Metabolome Database (HMDB), Metlin, KEGG compound, and PubChem, and further confirmed by experiments of fragmentation MS/MS and compared with authentic patterns. Differences in metabolytes among controls will be examined in metabolic pathways using KEGG and MassTrix web server.

***SUBPROJECT 5***

**efFeCtS of changes in the intestinal microbiota in the evolution of acute myocardial infarction in patients with diabetes and pre-diabetes**

**Summary**

Patients with acute coronary syndromes have high rates of abnormalities in the glucose metabolism, which includes the diagnosis of diabetes, pre-diabetes and stress hyperglycemia. Among those patients with metabolic syndrome, these alterations are present in approximately 75%^36^. Recently, changes in the intestinal microbiota in experimental model were associated with reversed phenotype of insulin resistance and obesity^140^. Furthermore, changes in microbiota were followed by expressive alteration in inflammatory parameters, including modulation of the T reg lymphocyte responses in visceral tissue. It is possible that obesity, microbiota, and insulin resistance change the balance of lymphocytes subtypes, affecting the atherosclerosis development and establishing a relationship between microbiota and atherosclerosis in obese patients with diabetes or pre-diabetes. Experimental study of our group showed that the induced atherosclerosis was deeply influenced by the presence of peritoneal inflammatory cells^10^. In this subproject the role microbiota will be examined in parameters of insulin resistance and metabolomics, as well as possible diferences in the amount of circulating lymphocytes subtypes, in patients with acute myocardial infarction.

**1. Proposal rationale**

More severe atherosclerosis in subjects with type 2 diabetes has been associated to multiples mechanisms, usually involving higher inflammatory activity, increased thrombotic risk, endothelial dysfunction, atherogenic dyslipidemia (small dense LDL, hypertriglyceridemia, and low HDL-C). These mechanisms of increased atherosclerosis in obese and sedentary subjects frequently are associated with abnormal fasting glucose, glucose intolerance or diagnosis of metabolic syndrome. The last condition has been associated with high cardiovascular mortality^141-151^. However, in the presence of pre-exhisting cardiovascular disease, the prognosis is still more serious. Currently, the role of intestinal microbiota in patients with type 2 diabetes or obesity has been highlighted.

Cani *et al*.^152^ proposed that lipopolisaccharides (LPS) from intestinal gram-negative bacteria can trigger inflammation. Continuously produced by cellular lysis, LPS are released to the blood stream under a high-fat diet.

Creely *et al*^153^ reported an increase in plasma LPS in subjects with type 2 diabetes compared to controls.

Toll-like receptors (TLR) appear to have a pivotal role in the activation of immune responses in mammals. TLR4 is activated by LPS and seems important in the inflammatory and immune reponses triggered by LPS, some of them related to the insulin resistance. Thus, TLR-4 has been proposed as a player of cross-talk between metabolic and inflammatory signalling^154^. The interaction of LPS and TLR4 is related to the release of pro-inflammatory cytokines such as TNF-α and IL-6^155^.

The TLRs and cytokines have also important role in the innate and adaptive immunity, affecting T cells, dendritic cells and the balance of Th1/Th2 phenotypes. The Th1 responses are characterized by an increase in IFN- α, IL-2 e IL-12, with this last cytokine related to the eliminations of intracellular pathogens and chronic metabolic diseases such as diabetes and obesity. Saturated fatty acids can also activate TLR-4 inducing the activation of inflammatory pathways, such as JNK e NF-*kappa*β in adipocytes and macrophages, related to insulin resistance and adiposity ^156^.

Increases in probiotic bacteria such as bifidobacteria may reduce circulating LPS, due to improvement in the intestinal barrier ^157^.

Thus, this subproject will examine patients with diagnosis of pre-diabetes and diabetes, since the acute phase of myocardial infarction. Diagnosis will follow the American Diabetes Association (ADA) criteria which is based on the glicated hemoglobin, due to the restrictions for other tests, such as the glucose tolerance or due to acute changes in glucose levels in patients with AMI^158^. Our hypothesis is that subjects with diabetes and pre-diabetes have more expressive imbalance between B lymphocyte subtypes related to obesity and intestinal microbiota composition. Our proposal includes the monitoring of the intestinal microbiota composition up to 6 months, and its association with subtypes of B lymphocytes, metabolomics and cMRI parameters. Other evaluations will be parameters of lipid metabolism and those related to insulin resistance. In these patients no interventions with pro- or antibiotics will be made.

**2. Objectives**

**2.1. General objectives**

2.1.1. To compare the microbiota composition in patients with pre-diabetes, diabetes and without diabetes or pre-diabetes at 30, 90, and 180 days after AMI.

2.1.2. To examine subtypes of lymphocytes at 30, 90, and 180 days after AMI.

2.1.3. To compare changes in the metabolomic analytes and possible associations with changes in the microbiota composition at 30, 90 and 180 days after AMI.

2.1.4. To compare cMRI parameters with microbiota composition.

**2.2. Secondary objectives**

2.2.1. To evaluate insulin resistance parameters (HOMA-IR) at 30, 90 and 180 dias.

2.2.2. Comparar entre os grupos modificações de dados antropométricos aos 30, 90, and 180 days after AMI.

**3. Methods**

- 1. Lymphocytes will be examined by flow-cytometry through specific CDs.
  2. Metabolomics, which will include specific analytes, such as the trimethylamine oxide (TMAO) by LC/MS-MS.
  3. HbA1c by HPLC.

3.4. Microbiota will be examined by KyberKompact Kyber plus, which allows the quantification of the main pathogenic or healthy bacteria.

*Study population*

In this subproject, patients will be included and classified as diabetes, pre-diabetes or no diabetes, according to the glycated hemoglobin (HbA1c) obtained in the first day of hospitalization. Pre-diabetes will be those with HbA1c between 5,7% to 6,4%, while diabetes those presenting HbA1c equal or greater of 6,5%, and no diabetes among those showing HbA1c <5,7%, according to the criteria of the 2012 American Diabetes Association (ADA). HbA1c will be examined by HPLC. Treatment of these patients will follow the recommendations of 2012 ADA for pharmacological therapies, and healthy lifestyle suggested by the National Cholesterol Education Program – Adult Treatment Panel (NCEP-ATP) III^2^.

Changes in the intestinal microbiota will be analized for possible associations with nutritional parameters, such as skinfold measurements, weight, BMI, abdominal circumference, food survey questionary, 24-hour reminder questionary, bioimpedance, and biochemical, metabolomic analysis, and subtypes of lymphocytes.

**3.** **Expected results**

The thematic project can identify subtypes of lymphocytes related to the evolution of ischemic cardiopathy. Our hypothesis is that an imbalance between subtypes of lymphocytes (higher number of B2 lymphocytes and lower of B1 lymphocytes beside increased participation of CD4 T lymphocytes) will be related to the classic risk factors, such as diabetes, obesity, sedentary lifestyle, hypertension and dyslipidemias). After the ischemic stimulus of acute myocardial infarction, higher presence of these lymphocytes, more inflammation and oxidized LDL will be associated with higher infarcted mass and progression of the ischemic cardiomyopathy due to differences in the reparative healing influenced by the exposure to subtypes of lymphocytes.

The lipid-lowering and antiplatelet strategies (notably rosuvastatin and ticagrelor) may minimize the muscle loss, due to their synergism in the improvement of endothelial function in the first hours after AMI. In addition, as simvastatin is a prodrug, it is possible that its pleiotropic effects may be smaller and delayed due to the interaction with clopidogrel, reducing pleiotropic effects (anti-inflammatory, antithrombotic and in the improvement of endothelial function) and due to lower effect of clopidogrel in the microcirculation (ticagrelor may increase adenosine).

The intestinal composition constitutes a new link between bacteria and atherosclerosis, as recent studies have shown LPS and metabolytes from our diet influencing inflammation, insulin resistance and destabilization of atherosclerotic plaques.

Analytes from lipidomics and metabolomics may identify metabolic pathways of importance in the ischemic cardiopathy, favoring future strategies. The better understanding and evaluation of the proposed therapies is one of the aims of this thematic project.

Studies involving the antibodies IgM and IgG against oxLDL will examine correlations with the cMRI parameters and with the subpopulations of lymphocytes analyzed.

Our Project also includes students of analytical chemistry and of biological área, allowing the establishment of a core of excellence, involving metabolomics and immunology, coordinated by professors with great expertise on these fields.

The study integrates four important public hospitals in Sao Paulo, *Hospital São Paulo – Universidade Federal de São Paulo, Instituto Dante Pazzanese de Cardiologia do Estado de São Paulo,* *Instituto do Coração* - *Universidade São Paulo*, *Santa Casa de Misericórdia de São Paulo*. In addition, integrates *Instituto de Química, Instituto de Física, Instituto de Ciências Biomédicas (all Universidade São Paulo)* and also integrates the Immunology and the Biophysics Departments of the *Universidade Federal de São Paulo.*

All these interfaces were the result of several seminars and meetings aimed at structuring the project, allowing the development of students, residents, postgraduated students, and scientists.

The study includes all precepts of good clinical practice and will be registered as clinical trial after its approval ([www.clinicaltrials.gov](http://www.clinicaltrials.gov)).

**4. SCIENTIFIC AND TECHNOLOGICAL CHALLENGES AND HOW TO OVERCOME**

The characterization of the human lymphocyte subtypes was a challenge in the beginning of the Project, but the scientists of the Immunology Department of UNIFESP quickly overcome these difficults in our pilot study.

The need of fresh samples for the studies such as those of flow-cytometry to determine microparticles and EPC will occur only in the Escola Paulista de Medicina e Instituto Dante Pazzanese de Cardiologia, due to the proximity of these institutions. There will cost reduction due to no need for a courrier. Other costs for the studies involving metabolomics, oxidized LDL, endothelial function, among others were reviewed and suitable to the sample size needed for these analyses.

The costs for the imaging (coronary angiography and cMRI) were debated in many meetings and these costs were minimized and adequate to the financial support of the study.

The drugs used in the study, which represented a considerable portion of the study’s resources will be a donation (principal investigator initiative). This donation will not be associated with participation in the study design, data access, publications or payment for the investigators.

Taking into account the sample size, initially we estimated the need of at least 7 patients included per month being the majority from the Unifesp.

A website will be created as well as the electronic medical record, by the IT department, including database and passords.

For monitoring and safety, a board with three physicians specialized in cardiology, imaging and epidemiology/statistics will be constituted and will analyze the study at each 1/3 of patients included. When necessary this independent board will notify the researchers the continuation of the study or its interruption.

1. **SCHEDULE FOR PROJECT EXECUTION**

|  | 2014 | 2015 | 2016 | 2017 | 2018 |
| --- | --- | --- | --- | --- | --- |
| Meetings for opening the centers  Patients recruitment  Meetings for monitoring of data  Meetings of specific groups*  Subproject 1  Subproject 2  Subproject 3  Subproject 4  Subproject 5 | **X**  **X**  **X**  **X**  **X**  **X**  **X**  **X** | **X**  **X**  **X**  **X**  **X**  **X**  **X**  **X** | **X**  **X**  **X**  **X**  **X**  **X**  **X**  **X** | **X**  **X**  **X**  **X**  **X**  **X**  **X**  **X** | **X**  **X**  **X**  **X**  **X**  **X**  **X** |

*Committee involving associated researchers and specific committees of MRI, immunology and lipidomics/metabolomics.

**6. ASSOCIATED RESEARCHERS**

*Adaptive and innate immunity*

Prof. Dr. José Daniel Lopes – *Departamento de Imunologia* EPM/UNIFESP

Prof. Dr. Mario Mariano – *Departamento de Imunologia* da EPM/UNIFESP

Profa. Dra.Ieda Longo Maugeri – *Departamento de Imunologia* EPM/UNIFESP

*Magnetic Resonance Imaging*

Prof. Dr. Gilberto Szarf – EPM/UNIFESP

Prof. Dr. Ibrahim Maschiarelli Pinto – *Instituto Dante Pazzanese de Cardiologia*

*Metabolomics/lipidomics*

Profa. Dra. Marina F. M. Tavares – *Instituto de Química* da USP

Dra. Aline Klassen – *Instituto de Química Campus Diadema* UNIFESP

*Oxidized lipoproteins and peptide synthesis*

Prof. Dra. Maria Cristina O Izar – *Disciplina de Cardiologia* EPM/UNIFESP

Prof. Dr. Magnus Gidlund – *Instituto de Ciências Biomédicas* – USP

Dra. Andrea Monteiro – *Instituto de Ciências Biomédicas* – USP

Prof. Dr. Antonio Martins Figueiredo – *Instituto de Física* da USP

Prof. Dr. Luiz A Juliano – *Departamento de Biofísica* UNIFESP

*Coronary angiography and interventions*

Prof. Dra. Claudia M R Alves - *Disciplina de Cardiologia* EPM/UNIFESP

Prof. Dr. Alexandre Abizaid – *Instituto Dante Pazzanese de Cardiologia*

Prof. Dr. Adriano Caixeta - *Disciplina de Cardiologia* EPM/UNIFESP

*Clinical trial/Coronary Unit*

Prof. Dr. Antonio C C Carvalho - *Disciplina de Cardiologia* EPM/UNIFESP

Prof. Dr. Rui Fernando Ramos – *Instituto Dante Pazzanese de Cardiologia* Prof. Prof. Dr. José Carlos Nicolau –*Instituto do Coração*/USP

Prof. Dr. Roberto Franken – *Santa Casa de Misericórdia de São Paulo*

*Independent data monitoring board*

Prof. Dr. Otavio Berwanger – cardiology/epidemiology– *Hospital do Coração* SP

Prof. Dr. Carlos Rochitte –cardiology/imaging - *Instituto do Coração*/USP

Prof. Dr. Luiz Antonio Machado Cesar – cardiology - *Instituto do Coração*/USP

*Information technology – database and electronic record*

Orlando Lima Cardoso – Director of the department of Information Technology – EPM/UNIFESP

**References:**

1. Fonseca FA, Paiva TB, Silva EG, *et al*. Atherosclerosis. 1998;139:237-42.
2. Fonseca FA, Izar MC, Fuster V, *et al*. Atherosclerosis. 2001;154:61-9.
3. Silva EP, Fonseca FA, Ihara SS, *et al*. J Cardiovasc Pharmacol. 2002;39:389-95.
4. Fonseca FA, Ihara SS, Izar MC, *et al*. Clin Exp Pharmacol Physiol. 2003;30:779-85.
5. Ferreira WP, Bertolami MC, Santos SN, *et al*. Pediatr Cardiol. 2007;28:8-13.
6. Monteiro CM, Pinheiro LF, Izar MC, *et al*. Braz J Med Biol Res. 2010;43:297-302.
7. da Silva EF, Fonseca FA, França CN, *et al*. AIDS. 2011;25:1595-601.
8. [França CN](https://www.ncbi.nlm.nih.gov/pubmed?term=Fran%C3%A7a%20CN%5BAuthor%5D&cauthor=true&cauthor_uid=22214900), [Pinheiro LF](https://www.ncbi.nlm.nih.gov/pubmed?term=Pinheiro%20LF%5BAuthor%5D&cauthor=true&cauthor_uid=22214900), [Izar MC](https://www.ncbi.nlm.nih.gov/pubmed?term=Izar%20MC%5BAuthor%5D&cauthor=true&cauthor_uid=22214900), *et al*. Circ J. 2012;76:729-36.
9. Pomaro DR, Ihara SS, Pinto LE, *et al*. J Cardiovasc Pharmacol. 2005;45:295-300.
10. Relvas WG, Izar MC, Segreto HR, *et al*. J Atheroscler Thromb. 2010;17:378-85.
11. Helfenstein T, Fonseca FA, Ihara SS, *et al*. Int J Exp Pathol. 2011;92:40-9.
12. [Feio CA, Izar MC, Ihara SS, *et al*.](http://www.ncbi.nlm.nih.gov/pubmed/22139433) J Atheroscler Thromb. 2011. [Epub ahead of print]
13. Albert MA, Glynn RJ, Fonseca FA, *et al*. Am Heart J. 2011;162:106-14.
14. de Lima Sanches P, de Mello MT, Elias N, *et al*. Hypertens Res. 2011;34:232-8.
15. Fonseca FA, França CN, Póvoa RM, Izar MC. Rev Neurol. 2010;51:551-60.
16. Ridker PM, MacFadyen JG, Fonseca FA, *et al*. Circ Cardiovasc Qual Outcomes. 2009;2:616-23.
17. Brandão SA, Izar MC, Fischer SM, *et al*. Am J Hypertens. 2010;23:208-14.
18. Fonseca FA, Izar MC. Expert Rev Cardiovasc Ther. 2009;7:1041-56.
19. Santos AO, Fonseca FA, Fischer SM, *et* al. Clin Chim Acta. 2009;406:113-8.
20. Glynn RJ, Danielson E, Fonseca FA, *et al*. N Engl J Med. 2009;360:1851-61.
21. Ridker PM, Danielson E, Fonseca FA, *et* al. Lancet. 2009;373:1175-82.
22. Ridker PM, Danielson E, Fonseca FA, *et al*. N Engl J Med. 2008;359:2195-207.
23. Ridker PM, Fonseca FA, Genest J, *et al*. Am J Cardiol. 2007;100:1659-64.
24. Ramos SC, Fonseca FA, Kasmas SH, *et al*. Nutr J. 2011;10:80.
25. Izar MC, Tegani DM, Kasmas SH, Fonseca FA. Genes Nutr. 2011;6:17-26.
26. Fonseca HA, Izar MC, Bianco HT, Fonseca FA. J Atheroscler Thromb. 2010;17:888.
27. Kasmas SH, Izar MC, França CN, *et al*. Braz J Med Biol Res 2012 (in press).
28. da Fonseca HA, Fonseca FA, Monteiro AM, *et al*. Int J Cardiol. 2012;157:131-3.
29. Izar MC, Fonseca HA, *et al.* Diab Vasc Dis Res. 2012. [Epub ahead of print].
30. [Colossimo AP, Costa Fde A, Riera AR, *et al.*](http://www.ncbi.nlm.nih.gov/pubmed/21845342) Arq Bras Cardiol. 2011;97:225-31.
31. Costa F de A, Bombig MT, de Lima VC, *et al*. Int J Cardiol. 2011;151:374-5.
32. Marui FR, Bombig MT, Francisco YA, *et al*. Arq Bras Cardiol. 2010;95:536-40.
33. Santos MA, Costa Fde A, Travessa AF, *et al*. Arq Bras Cardiol. 2010;94:620-4.
34. Schwartz GG, Olsson AG, Ballantyne CM, *et al*. Am Heart J. 2009;158:896-901.
35. Brollo L, Bombig MT, Mazzaro CL, *et al*. Arq Bras Cardiol. 2009;92:351-5.
36. Monteiro CM, Oliveira L, Izar MC, *et al*. Arq Bras Cardiol. 2009;92:89-99.
37. da Costa W, Riera AR, Costa Fde A, *et al*. J Electrocardiol. 2008;41:724-9.
38. Izar MC, Helfenstein T, Ihara SS,*et al*. Atherosclerosis. 2009;204:165-70.
39. Mazzaro C do L, Costa F de A, Bombig MT, *et al*. Arq Bras Cardiol. 2008;90:227-31.
40. Sposito AC, Caramelli B, Fonseca FA, *et al*. Arq Bras Cardiol. 2007;88 Suppl 1:2-19.
41. Teixeira M, Kasinski N, Izar MC, *et al*. Arq Bras Cardiol. 2006;87:3-11.
42. Middleton A, Binbrek AS, Fonseca FA, *et al*. Curr Med Res Op. 2006;22:1181-91.
43. Fonseca FA, Izar MC, Silva MA, *et al.* Evid Based Cardiovasc Med. 2006;10:96-100.
44. Mendes GA, Martinez TL, Izar MC, *et al*. Arq Bras Cardiol. 2006;86:361-5.
45. Back Giuliano I, Caramelli B, Pellanda L, *et al*. Arq Bras Cardiol. 2005;85:4-36.
46. Fonseca FA. Arq Bras Cardiol. 2005 Oct;85 Suppl 5:9-14.
47. Fonseca FA, Ruiz A, Cardona-Muñoz EG, *et al* Curr Med Res Opin. 2005;21:1307-15.
48. Helfenstein T, Fonseca FA, Relvas WG, *et al*. Clin Chim Acta. 2005;355:165-72.
49. Relvas WG, Izar MC, Helfenstein T, *et al*. Atherosclerosis. 2005;178:101-5.
50. Fonseca FA, Izar MC. Arq Bras Cardiol. 2004;83:371-2.
51. Elias MC, Bolívar MS, Fonseca FA, *et al*. Arq Bras Cardiol. 2004;82:143-6.
52. Feio CM, Fonseca FA, Rego SS, *et al*. Arq Bras Cardiol. 2003;81:596-9.
53. Bricarello LP, Kasinski N, Bertolami MC, *et al*. Nutrition. 2004;20:200-4.
54. Izar MC, Fonseca FA, Ihara SS, *et al*. [.](http://www.ncbi.nlm.nih.gov/pubmed/12754559)Arq Bras Cardiol. 2003;80:379-95.
55. Santos RD, Spósito AC, dos Santos JE, *et al*. Arq Bras Cardiol. 2000;75:289-302.
56. Fonseca FA, Novazzi JP, Cendoroglo MS, *et al*. Arq Bras Cardiol. 1996;66:33-5.
57. Novazzi JP, Fonseca FA, Feres MC, *et al*. Arq Bras Cardiol. 1994;62:395-8.
58. de Carvalho VB, Fonseca FA, *et al*. Arq Bras Cardiol. 1991;57:41-5.
59. [Pinheiro LF](https://www.ncbi.nlm.nih.gov/pubmed?term=Pinheiro%20LF%5BAuthor%5D&cauthor=true&cauthor_uid=22569318), [França CN](https://www.ncbi.nlm.nih.gov/pubmed?term=Fran%C3%A7a%20CN%5BAuthor%5D&cauthor=true&cauthor_uid=22569318), [Izar MC](https://www.ncbi.nlm.nih.gov/pubmed?term=Izar%20MC%5BAuthor%5D&cauthor=true&cauthor_uid=22569318), *et al.* Int J Cardiol 2012;158:125-8.
60. Pereira IA, Borba EF, 2008. Swiss Med. Wkly 2008;138:534–9.
61. Moore KJ, Tabas I. Cell 2011;145:341–55.
62. Weber C, *et al.* J Clin Invest 2011;121:2898–910.
63. Vitale G, *et al*. Mol. Immunol. 2010;48:1–8.
64. Miller YI, *et al*. Circ Res 2011;108:235-48.
65. Galkina E.*et al*. J Exp Med 2006;203:1273–82.
66. Ponnuswamy P, Van Vré EA, Mallat Z, Tedgui A. Vasc Pharmacol 2012;56:193-203.
67. van Gils JM, Derby MC, Fernandes LR, *et al*. Nat Immunol 2012;13:136-43.
68. Campbell KA, Lipinski MJ, Doran AC, *et al*. Circ Res 2012;110:889-900.
69. Gerszten RE, Tager AM. N Engl J Med 2012;366:1734-6.
70. Goodchild TT, Robinson KA PHD, *et al*. JACC cardiovasc Interv 2009; 2:1005–16.
71. Dutta P, Courties G, Wei Y, *et al*. Nature 2012 (Epub ahead of print).
72. Moreira FT, Ramos SC, Monteiro AM, et al. Life Sci 2014;98:83-7
73. Hilgendorf I, Theurl I, Gerhardt LMS, et al. Circulation 2014;129:1677-87.
74. Kyaw T, Toy C, Krishnamurth S. Circ Res 2011;109:830-40.
75. [Schwartz GG](http://www.ncbi.nlm.nih.gov/pubmed?term=Schwartz%20GG%5BAuthor%5D&cauthor=true&cauthor_uid=11277825), [Olsson AG](http://www.ncbi.nlm.nih.gov/pubmed?term=Olsson%20AG%5BAuthor%5D&cauthor=true&cauthor_uid=11277825), [Ezekowitz MD](http://www.ncbi.nlm.nih.gov/pubmed?term=Ezekowitz%20MD%5BAuthor%5D&cauthor=true&cauthor_uid=11277825), *et al*. JAMA. 2001;285:1711-8.
76. [Cannon CP](http://www.ncbi.nlm.nih.gov/pubmed?term=Cannon%20CP%5BAuthor%5D&cauthor=true&cauthor_uid=15007110), [Braunwald E](http://www.ncbi.nlm.nih.gov/pubmed?term=Braunwald%20E%5BAuthor%5D&cauthor=true&cauthor_uid=15007110), [McCabe CH](http://www.ncbi.nlm.nih.gov/pubmed?term=McCabe%20CH%5BAuthor%5D&cauthor=true&cauthor_uid=15007110), *et al*. N Engl J Med. 2004;350:1495-504.
77. [Spencer FA](http://www.ncbi.nlm.nih.gov/pubmed?term=Spencer%20FA%5BAuthor%5D&cauthor=true&cauthor_uid=15505131), [Fonarow GC](http://www.ncbi.nlm.nih.gov/pubmed?term=Fonarow%20GC%5BAuthor%5D&cauthor=true&cauthor_uid=15505131), [Frederick PD](http://www.ncbi.nlm.nih.gov/pubmed?term=Frederick%20PD%5BAuthor%5D&cauthor=true&cauthor_uid=15505131), *et al*. Arch Intern Med. 2004;164:2162-8.
78. [Fonarow GC](http://www.ncbi.nlm.nih.gov/pubmed?term=Fonarow%20GC%5BAuthor%5D&cauthor=true&cauthor_uid=16125480), [Wright RS](http://www.ncbi.nlm.nih.gov/pubmed?term=Wright%20RS%5BAuthor%5D&cauthor=true&cauthor_uid=16125480), [Spencer FA](http://www.ncbi.nlm.nih.gov/pubmed?term=Spencer%20FA%5BAuthor%5D&cauthor=true&cauthor_uid=16125480), *et al.* Am J Cardiol. 2005;96:611-6.
79. [Wright RS](http://www.ncbi.nlm.nih.gov/pubmed?term=Wright%20RS%5BAuthor%5D&cauthor=true&cauthor_uid=15975672), [Bybee K](http://www.ncbi.nlm.nih.gov/pubmed?term=Bybee%20K%5BAuthor%5D&cauthor=true&cauthor_uid=15975672), [Miller WL](http://www.ncbi.nlm.nih.gov/pubmed?term=Miller%20WL%5BAuthor%5D&cauthor=true&cauthor_uid=15975672), *et al*. Int J Cardiol. 2006;108:314-9.
80. [Di Sciascio G](http://www.ncbi.nlm.nih.gov/pubmed?term=Di%20Sciascio%20G%5BAuthor%5D&cauthor=true&cauthor_uid=19643320), [Patti G](http://www.ncbi.nlm.nih.gov/pubmed?term=Patti%20G%5BAuthor%5D&cauthor=true&cauthor_uid=19643320), [Pasceri V](http://www.ncbi.nlm.nih.gov/pubmed?term=Pasceri%20V%5BAuthor%5D&cauthor=true&cauthor_uid=19643320), *et al*. J Am Coll Cardiol. 2009;54:558-65.
81. [Yun KH](http://www.ncbi.nlm.nih.gov/pubmed?term=Yun%20KH%5BAuthor%5D&cauthor=true&cauthor_uid=20471117), [Oh SK](http://www.ncbi.nlm.nih.gov/pubmed?term=Oh%20SK%5BAuthor%5D&cauthor=true&cauthor_uid=20471117), [Rhee SJ](http://www.ncbi.nlm.nih.gov/pubmed?term=Rhee%20SJ%5BAuthor%5D&cauthor=true&cauthor_uid=20471117), *et al*. Int J Cardiol. 2011;146:68-72.
82. [Gurbel PA](https://www.ncbi.nlm.nih.gov/pubmed?term=Gurbel%20PA%5BAuthor%5D&cauthor=true&cauthor_uid=19923168), [Bliden KP](https://www.ncbi.nlm.nih.gov/pubmed?term=Bliden%20KP%5BAuthor%5D&cauthor=true&cauthor_uid=19923168), [Butler K](https://www.ncbi.nlm.nih.gov/pubmed?term=Butler%20K%5BAuthor%5D&cauthor=true&cauthor_uid=19923168), *et al.* Circulation. 2009;120:2577-85.
83. [Wallentin L](https://www.ncbi.nlm.nih.gov/pubmed?term=Wallentin%20L%5BAuthor%5D&cauthor=true&cauthor_uid=19717846), [Becker RC](https://www.ncbi.nlm.nih.gov/pubmed?term=Becker%20RC%5BAuthor%5D&cauthor=true&cauthor_uid=19717846), [Budaj A](https://www.ncbi.nlm.nih.gov/pubmed?term=Budaj%20A%5BAuthor%5D&cauthor=true&cauthor_uid=19717846), *et al.* N Engl J Med. 2009;361:1045-57.
84. Wiviott SD, Braunwald E, McCabe CH, *et al*. N Engl J Med. 2007;357:2001-15.
85. [Serebruany VL](https://www.ncbi.nlm.nih.gov/pubmed?term=Serebruany%20VL%5BAuthor%5D&cauthor=true&cauthor_uid=21212672). Cardiology. 2010;117:231-3.
86. [Serebruany VL](https://www.ncbi.nlm.nih.gov/pubmed?term=Serebruany%20VL%5BAuthor%5D&cauthor=true&cauthor_uid=20024505). Thromb Haemost. 2010;103:259-61.
87. [Serebruany VL](https://www.ncbi.nlm.nih.gov/pubmed?term=Serebruany%20VL%5BAuthor%5D&cauthor=true&cauthor_uid=20007979), [Atar D](https://www.ncbi.nlm.nih.gov/pubmed?term=Atar%20D%5BAuthor%5D&cauthor=true&cauthor_uid=20007979). Eur Heart J. 2010;31:764-7.
88. [Grzesk G](https://www.ncbi.nlm.nih.gov/pubmed?term=Grzesk%20G%5BAuthor%5D&cauthor=true&cauthor_uid=22265722), [Kozinski M](https://www.ncbi.nlm.nih.gov/pubmed?term=Kozinski%20M%5BAuthor%5D&cauthor=true&cauthor_uid=22265722), [Navarese EP](https://www.ncbi.nlm.nih.gov/pubmed?term=Navarese%20EP%5BAuthor%5D&cauthor=true&cauthor_uid=22265722), *et al*. Thromb Res. 2012. [Epub ahead of print].
89. [Braunwald E](https://www.ncbi.nlm.nih.gov/pubmed?term=Braunwald%20E%5BAuthor%5D&cauthor=true&cauthor_uid=6754130), [Kloner RA](https://www.ncbi.nlm.nih.gov/pubmed?term=Kloner%20RA%5BAuthor%5D&cauthor=true&cauthor_uid=6754130). Circulation. 1982;66:1146-9.
90. [Rahimtoola SH](https://www.ncbi.nlm.nih.gov/pubmed?term=Rahimtoola%20SH%5BAuthor%5D&cauthor=true&cauthor_uid=2783527). Am Heart J. 1989;117:211-21.
91. [Saraste A](https://www.ncbi.nlm.nih.gov/pubmed?term=Saraste%20A%5BAuthor%5D&cauthor=true&cauthor_uid=18242487), [Nekolla S](https://www.ncbi.nlm.nih.gov/pubmed?term=Nekolla%20S%5BAuthor%5D&cauthor=true&cauthor_uid=18242487), [Schwaiger M](https://www.ncbi.nlm.nih.gov/pubmed?term=Schwaiger%20M%5BAuthor%5D&cauthor=true&cauthor_uid=18242487). J Nucl Cardiol. 2008;15:105-17.
92. [Roes SD](https://www.ncbi.nlm.nih.gov/pubmed?term=Roes%20SD%5BAuthor%5D&cauthor=true&cauthor_uid=17826372), [Kelle S](https://www.ncbi.nlm.nih.gov/pubmed?term=Kelle%20S%5BAuthor%5D&cauthor=true&cauthor_uid=17826372), [Kaandorp TA](https://www.ncbi.nlm.nih.gov/pubmed?term=Kaandorp%20TA%5BAuthor%5D&cauthor=true&cauthor_uid=17826372), *et al.* Am J Cardiol. 2007;100:930-6.
93. [Bello D](https://www.ncbi.nlm.nih.gov/pubmed?term=Bello%20D%5BAuthor%5D&cauthor=true&cauthor_uid=15808771), [Fieno DS](https://www.ncbi.nlm.nih.gov/pubmed?term=Fieno%20DS%5BAuthor%5D&cauthor=true&cauthor_uid=15808771), [Kim RJ](https://www.ncbi.nlm.nih.gov/pubmed?term=Kim%20RJ%5BAuthor%5D&cauthor=true&cauthor_uid=15808771), *et al*. J Am Coll Cardiol. 2005;45:1104-8.
94. [Kim RJ](https://www.ncbi.nlm.nih.gov/pubmed?term=Kim%20RJ%5BAuthor%5D&cauthor=true&cauthor_uid=11078769), [Wu E](https://www.ncbi.nlm.nih.gov/pubmed?term=Wu%20E%5BAuthor%5D&cauthor=true&cauthor_uid=11078769), [Rafael A](https://www.ncbi.nlm.nih.gov/pubmed?term=Rafael%20A%5BAuthor%5D&cauthor=true&cauthor_uid=11078769), *et al*. N Engl J Med. 2000;343:1445-53.
95. Cerqueira MD, Weissman NJ, Dilsizian V, *et al*. Circulation 2002;105:539-42.
96. Kramer CM. Cardiol Clin 1998;16:267-76.
97. Gudmundsson P, Winter R, Dencker M, *et al*. Clin Physiol Funct Imag 2006;26:32-8.
98. Stork A, Muellerleile K, Bansmann PM, *et al*. Eur Radiol 2007;17:610-7.
99. KnoppMV, Schoenberg SO, Rehm C, *et al*. Invest Radiol 2002;37:706-15.

100. McCrohon JA, Moon JC, Prasad SK, *et al*. Circulation 2003;108:54-9

101. Vohringer M, Mahrholdt H, Yilmaz A, Sechtem U. Herz 2007;32:129-37.

102. Mahrholdt H, Wagner A, Judd RM, *et al*. Eur Heart J 2005;26:1461-74.

103. Weinsaft JW, Klem I,Judd RM. Cardiol Clin 2007;25:35-56.

104. Sparrow P, Messroghli DR, Reid S, *et al*. Am J Radiol 2006;187:W630–W635.

105. [Camici GG](http://www.ncbi.nlm.nih.gov/pubmed?term=Camici%20GG%5BAuthor%5D&cauthor=true&cauthor_uid=19434051), [Sudano I](http://www.ncbi.nlm.nih.gov/pubmed?term=Sudano%20I%5BAuthor%5D&cauthor=true&cauthor_uid=19434051), [Noll G](http://www.ncbi.nlm.nih.gov/pubmed?term=Noll%20G%5BAuthor%5D&cauthor=true&cauthor_uid=19434051), *et al*. Curr Opin Nephrol Hypertens. 2009;18:134-7.

106. [Werner N](http://www.ncbi.nlm.nih.gov/pubmed?term=Werner%20N%5BAuthor%5D&cauthor=true&cauthor_uid=17453672), [Nickenig G](http://www.ncbi.nlm.nih.gov/pubmed?term=Nickenig%20G%5BAuthor%5D&cauthor=true&cauthor_uid=17453672). Ann Med. 2007;39:82-90.

107. [Boulanger CM](http://www.ncbi.nlm.nih.gov/pubmed?term=Boulanger%20CM%5BAuthor%5D&cauthor=true&cauthor_uid=16801490), [Amabile N](http://www.ncbi.nlm.nih.gov/pubmed?term=Amabile%20N%5BAuthor%5D&cauthor=true&cauthor_uid=16801490), [Tedgui A](http://www.ncbi.nlm.nih.gov/pubmed?term=Tedgui%20A%5BAuthor%5D&cauthor=true&cauthor_uid=16801490). Hypertension. 2006;48:180-6.

108. [Quyyumi AA](http://www.ncbi.nlm.nih.gov/pubmed?term=Quyyumi%20AA%5BAuthor%5D&cauthor=true&cauthor_uid=21167340), [Waller EK](http://www.ncbi.nlm.nih.gov/pubmed?term=Waller%20EK%5BAuthor%5D&cauthor=true&cauthor_uid=21167340), [Murrow J](http://www.ncbi.nlm.nih.gov/pubmed?term=Murrow%20J%5BAuthor%5D&cauthor=true&cauthor_uid=21167340), *et al*. Am Heart J. 2011;161:98-105.

109. Geisler T, Fekecs L, Wurster T, *et al*. Eur J Radiol. 2012;81:e486-90.

110. Sibbing D, Braun S, Morath T, *et al*. J Am Coll Cardiol 2009; 53: 849-56.

111. [Gori T](http://www.ncbi.nlm.nih.gov/pubmed?term=Gori%20T%5BAuthor%5D&cauthor=true&cauthor_uid=21920964), [Muxel S](http://www.ncbi.nlm.nih.gov/pubmed?term=Muxel%20S%5BAuthor%5D&cauthor=true&cauthor_uid=21920964), [Damaske A](http://www.ncbi.nlm.nih.gov/pubmed?term=Damaske%20A%5BAuthor%5D&cauthor=true&cauthor_uid=21920964), *et al*. Eur Heart J. 2012;33:363-71.

112. [Spiro JR](http://www.ncbi.nlm.nih.gov/pubmed?term=Spiro%20JR%5BAuthor%5D&cauthor=true&cauthor_uid=21037253), [Digby JE](http://www.ncbi.nlm.nih.gov/pubmed?term=Digby%20JE%5BAuthor%5D&cauthor=true&cauthor_uid=21037253), [Ghimire G](http://www.ncbi.nlm.nih.gov/pubmed?term=Ghimire%20G%5BAuthor%5D&cauthor=true&cauthor_uid=21037253), *et al*. Eur Heart J. 2011;32:856-66.

113. Eshtehardi P, Windecker S, Cook S, *et al*. Am Heart J 2010; 159: 891-8.

114. [Breet NJ](http://www.ncbi.nlm.nih.gov/pubmed?term=Breet%20NJ%5BAuthor%5D&cauthor=true&cauthor_uid=20179285), [van Werkum JW](http://www.ncbi.nlm.nih.gov/pubmed?term=van%20Werkum%20JW%5BAuthor%5D&cauthor=true&cauthor_uid=20179285), [Bouman HJ](http://www.ncbi.nlm.nih.gov/pubmed?term=Bouman%20HJ%5BAuthor%5D&cauthor=true&cauthor_uid=20179285), et al. [JAMA](http://www.ncbi.nlm.nih.gov/pubmed/20179285) 2010;303:754-62.

115. [Libby P](http://www.ncbi.nlm.nih.gov/pubmed?term=Libby%20P%5BAuthor%5D&cauthor=true&cauthor_uid=19942084), [Ridker PM](http://www.ncbi.nlm.nih.gov/pubmed?term=Ridker%20PM%5BAuthor%5D&cauthor=true&cauthor_uid=19942084), [Hansson GK](http://www.ncbi.nlm.nih.gov/pubmed?term=Hansson%20GK%5BAuthor%5D&cauthor=true&cauthor_uid=19942084). J Am Coll Cardiol. 2009;54:2129-38.

116. [Hansson GK](http://www.ncbi.nlm.nih.gov/pubmed?term=Hansson%20GK%5BAuthor%5D&cauthor=true&cauthor_uid=21321594), [Hermansson A](http://www.ncbi.nlm.nih.gov/pubmed?term=Hermansson%20A%5BAuthor%5D&cauthor=true&cauthor_uid=21321594). Nat Immunol. 2011;12:204-12.

117. [Libby P](http://www.ncbi.nlm.nih.gov/pubmed?term=Libby%20P%5BAuthor%5D&cauthor=true&cauthor_uid=21593864), [Ridker PM](http://www.ncbi.nlm.nih.gov/pubmed?term=Ridker%20PM%5BAuthor%5D&cauthor=true&cauthor_uid=21593864), [Hansson GK](http://www.ncbi.nlm.nih.gov/pubmed?term=Hansson%20GK%5BAuthor%5D&cauthor=true&cauthor_uid=21593864). Nature. 2011;473:317-25.

118. Goodchild TT, Robinson KA, *et al*. JACC Cardiovasc Interv. 2009;2:1005-16.

119. [Hofmann U](http://www.ncbi.nlm.nih.gov/pubmed?term=Hofmann%20U%5BAuthor%5D&cauthor=true&cauthor_uid=22388323), [Beyersdorf N](http://www.ncbi.nlm.nih.gov/pubmed?term=Beyersdorf%20N%5BAuthor%5D&cauthor=true&cauthor_uid=22388323), [Weirather J](http://www.ncbi.nlm.nih.gov/pubmed?term=Weirather%20J%5BAuthor%5D&cauthor=true&cauthor_uid=22388323), *et al*. Circulation. 2012;125:1652-63.

120. [Campbell KA](http://www.ncbi.nlm.nih.gov/pubmed?term=Campbell%20KA%5BAuthor%5D&cauthor=true&cauthor_uid=22427326), [Lipinski MJ](http://www.ncbi.nlm.nih.gov/pubmed?term=Lipinski%20MJ%5BAuthor%5D&cauthor=true&cauthor_uid=22427326), [Doran AC](http://www.ncbi.nlm.nih.gov/pubmed?term=Doran%20AC%5BAuthor%5D&cauthor=true&cauthor_uid=22427326), *et al*. Circ Res. 2012;110:889-900.

121. [Gerszten RE](http://www.ncbi.nlm.nih.gov/pubmed?term=Gerszten%20RE%5BAuthor%5D&cauthor=true&cauthor_uid=22551134), [Tager AM](http://www.ncbi.nlm.nih.gov/pubmed?term=Tager%20AM%5BAuthor%5D&cauthor=true&cauthor_uid=22551134). N Engl J Med. 2012;366:1734-6.

122. Bernal-Mizrachi L, Jy W, Jimenez JJ, [Pastor J](http://www.ncbi.nlm.nih.gov/pubmed?term=%22Pastor%20J%22%5BAuthor%5D), *et. al*. Am Heart J. 2003;145:962-70.

123. [van Gils JM](http://www.ncbi.nlm.nih.gov/pubmed?term=van%20Gils%20JM%5BAuthor%5D&cauthor=true&cauthor_uid=22231519), [Derby MC](http://www.ncbi.nlm.nih.gov/pubmed?term=Derby%20MC%5BAuthor%5D&cauthor=true&cauthor_uid=22231519), [Fernandes LR](http://www.ncbi.nlm.nih.gov/pubmed?term=Fernandes%20LR%5BAuthor%5D&cauthor=true&cauthor_uid=22231519), *et al.* Nat Immunol. 2012;13:136-43.

124. [Kyaw T](http://www.ncbi.nlm.nih.gov/pubmed?term=Kyaw%20T%5BAuthor%5D&cauthor=true&cauthor_uid=21868694), [Tay C](http://www.ncbi.nlm.nih.gov/pubmed?term=Tay%20C%5BAuthor%5D&cauthor=true&cauthor_uid=21868694), [Krishnamurthi S](http://www.ncbi.nlm.nih.gov/pubmed?term=Krishnamurthi%20S%5BAuthor%5D&cauthor=true&cauthor_uid=21868694), *et al*. Circ Res. 2011;109:830-40.

125. [Kyaw T](http://www.ncbi.nlm.nih.gov/pubmed?term=Kyaw%20T%5BAuthor%5D&cauthor=true&cauthor_uid=21881498), [Tipping P](http://www.ncbi.nlm.nih.gov/pubmed?term=Tipping%20P%5BAuthor%5D&cauthor=true&cauthor_uid=21881498), [Toh BH](http://www.ncbi.nlm.nih.gov/pubmed?term=Toh%20BH%5BAuthor%5D&cauthor=true&cauthor_uid=21881498), [Bobik A](http://www.ncbi.nlm.nih.gov/pubmed?term=Bobik%20A%5BAuthor%5D&cauthor=true&cauthor_uid=21881498). Curr Opin Lipidol. 2011;22:373-9.

126. [Kyaw](http://www.ncbi.nlm.nih.gov/sites/entrez?cmd=search&db=PubMed&term=%20Kyaw%2BT%5Bauth%5D) T, [Tay](http://www.ncbi.nlm.nih.gov/sites/entrez?cmd=search&db=PubMed&term=%20Tay%2BC%5Bauth%5D) C, [Hosseini](http://www.ncbi.nlm.nih.gov/sites/entrez?cmd=search&db=PubMed&term=%20Hosseini%2BH%5Bauth%5D) H, *et al*. PLoS One. 2012;7:e29371.

127. Instituto Nacional de Fluidos Complexos – INCT-FCx. <http://fluidos.usp.br/>

128. [Monteiro AM](http://www.ncbi.nlm.nih.gov/pubmed?term=Monteiro%20AM%5BAuthor%5D&cauthor=true&cauthor_uid=19254121), [Jardini MA](http://www.ncbi.nlm.nih.gov/pubmed?term=Jardini%20MA%5BAuthor%5D&cauthor=true&cauthor_uid=19254121), [Alves S](http://www.ncbi.nlm.nih.gov/pubmed?term=Alves%20S%5BAuthor%5D&cauthor=true&cauthor_uid=19254121), *et al*. J Periodontol. 2009;80:378-88.

129. Abonnenc M, Stegemann C, Mayr M. Expert Rev Proteomics 2010;7:811–13.

130. Lewis GD, Asnani A, Gerszten RE. J Am Coll Cardiol. 2008;52:117-23.

131. Cavill R, Keun HC, Holmes E, *et al.* Bioinformatics 2008, 25:112-8.

132. Watson AD. J Lipid Res 2006, 47:2101-2111.

133. Lewis GD, Wei R, Liu E et al. J Clin Invest 2008;118: 3503–12.

134. [Wu DJ](http://www.ncbi.nlm.nih.gov/pubmed?term=Wu%20DJ%5BAuthor%5D&cauthor=true&cauthor_uid=22040517), [Zhu BJ](http://www.ncbi.nlm.nih.gov/pubmed?term=Zhu%20BJ%5BAuthor%5D&cauthor=true&cauthor_uid=22040517), [Wang XD](http://www.ncbi.nlm.nih.gov/pubmed?term=Wang%20XD%5BAuthor%5D&cauthor=true&cauthor_uid=22040517). J Clin Bioinform 2011;1:30.

135. Martin JC, Canlet C, Delplanque B. Atherosclerosis 2009, 206:127-33.

136. Zhang F, Jia Z, Gao P. Talanta 2009, 79:836-844.

137. Mayr M, Chung YL, Mayr U. ATVB 2005, 25:2135-42.

138. Li N, Liu JY, Timofeyev V, *et al*. J Mol Cell Cardiol.2009;47:835-45.

139. Liu JY, Yang J, Inceoglu B, *et al*. Biochem Pharmacol. 2010;79:880-7.

140. Bligh EG, Dyer WJ. Can J Biochem Physiol. 1959;37:911-7.

141. Miyazaki H, Ishibashi M, Takayama H, *et al*. J Chromatogr 1984;289:249-58.

142. Ferreira CR, Saraiva SA, Catharino RR, *et al.* J Lipid Res. 2010;51:1218-27.

143. Saric J, Want EJ, Duthaler U, *et al.* Anal. Chem. 2012, 84: 6963−6972.

144. Trygg J, Holmes E, Lundstedt T. J. Proteome Res. 2007, 6: 469-479.

145. [Caricilli AM](http://www.ncbi.nlm.nih.gov/pubmed?term=Caricilli%20AM%5BAuthor%5D&cauthor=true&cauthor_uid=22162948), [Picardi PK](http://www.ncbi.nlm.nih.gov/pubmed?term=Picardi%20PK%5BAuthor%5D&cauthor=true&cauthor_uid=22162948), [de Abreu LL](http://www.ncbi.nlm.nih.gov/pubmed?term=de%20Abreu%20LL%5BAuthor%5D&cauthor=true&cauthor_uid=22162948), *et al*. PLoS Biol. 2011;9:e1001212.

146. [Unwin N](http://www.ncbi.nlm.nih.gov/pubmed?term=Unwin%20N%5BAuthor%5D&cauthor=true&cauthor_uid=12207806), [Shaw J](http://www.ncbi.nlm.nih.gov/pubmed?term=Shaw%20J%5BAuthor%5D&cauthor=true&cauthor_uid=12207806), [Zimmet P](http://www.ncbi.nlm.nih.gov/pubmed?term=Zimmet%20P%5BAuthor%5D&cauthor=true&cauthor_uid=12207806), [Alberti KG](http://www.ncbi.nlm.nih.gov/pubmed?term=Alberti%20KG%5BAuthor%5D&cauthor=true&cauthor_uid=12207806). Diabet Med. 2002;19:708-23.

147. [Lakka HM](http://www.ncbi.nlm.nih.gov/pubmed?term=Lakka%20HM%5BAuthor%5D&cauthor=true&cauthor_uid=12460094), [Laaksonen DE](http://www.ncbi.nlm.nih.gov/pubmed?term=Laaksonen%20DE%5BAuthor%5D&cauthor=true&cauthor_uid=12460094), [Lakka TA](http://www.ncbi.nlm.nih.gov/pubmed?term=Lakka%20TA%5BAuthor%5D&cauthor=true&cauthor_uid=12460094), *et al*. JAMA. 2002;288:2709-16.

148. [Wang J](http://www.ncbi.nlm.nih.gov/pubmed?term=Wang%20J%5BAuthor%5D&cauthor=true&cauthor_uid=17303589), [Ruotsalainen S](http://www.ncbi.nlm.nih.gov/pubmed?term=Ruotsalainen%20S%5BAuthor%5D&cauthor=true&cauthor_uid=17303589), [Moilanen L](http://www.ncbi.nlm.nih.gov/pubmed?term=Moilanen%20L%5BAuthor%5D&cauthor=true&cauthor_uid=17303589), *et al*. Eur Heart J. 2007;28:857-64.

149. [Malik S](http://www.ncbi.nlm.nih.gov/pubmed?term=Malik%20S%5BAuthor%5D&cauthor=true&cauthor_uid=15326067), [Wong ND](http://www.ncbi.nlm.nih.gov/pubmed?term=Wong%20ND%5BAuthor%5D&cauthor=true&cauthor_uid=15326067), [Franklin SS](http://www.ncbi.nlm.nih.gov/pubmed?term=Franklin%20SS%5BAuthor%5D&cauthor=true&cauthor_uid=15326067), *et al.* Circulation. 2004;110:1245-50.

150. Vamos EP, Millett C, Parsons C, *et al*. Diabetes Care. 2012;35:265-72.

151. Nicolau JC, Serrano CV Jr, Giraldez RR, *et al*. Diabetes Care. 2012;35:150-2.

152. Cani PD, Bibiloni R, Knauf C, *et al*. Diabetes; 2008, 57: 1470-81.

153. Creely SJ, McTernan PG, *et al*. Am J Physiol Endocrinol Metab. 2007, 292: E740-47.

154. Tsukumo DML, Carvalho-Filho MA, *et al*. Diabetes. 2007, 56: 1986-1998.

155. Hattori M, Taylor TD. DNA Research. 2009;16:1-12.

156. Haiat PD. Obesidade e intestino. In: Naves A. Nutrição Clínica Funcional – Obesidade. VP Editora. São Paulo. 2009.

157. Kort S, Keszthelyi D, Masclee AAM. Obesity reviews. 2011, 12: 449-58.

158. [American Diabetes Association](http://www.ncbi.nlm.nih.gov/pubmed?term=American%20Diabetes%20Association%5BCorporate%20Author%5D&cauthor=true&cauthor_uid=22187472). Diabetes Care. 2012;35:S64-71.
